# Advances and Perspectives in Tissue Culture and Genetic Engineering of Cannabis

**DOI:** 10.3390/ijms22115671

**Published:** 2021-05-26

**Authors:** Mohsen Hesami, Austin Baiton, Milad Alizadeh, Marco Pepe, Davoud Torkamaneh, Andrew Maxwell Phineas Jones

**Affiliations:** 1 Department of Plant Agriculture, University of Guelph, Guelph, ON N1G 2W1, Canada; mhesami@uoguelph.ca (M.H.); abaiton@uoguelph.ca (A.B.); pepem@uoguelph.ca (M.P.); 2 Department of Botany, University of British Columbia, Vancouver, BC V6T 1Z4, Canada; milad.alizadeh@botany.ubc.ca; 3 Département de Phytologie, Université Laval, Québec City, QC G1V 0A6, Canada; davoud.torkamaneh.1@ulaval.ca

**Keywords:** haploid production, hemp, gene transformation, genome editing, in vitro culture, marijuana, morphogenic genes, organogenesis, somatic embryogenesis, polyploidy

## Abstract

For a long time, *Cannabis sativa* has been used for therapeutic and industrial purposes. Due to its increasing demand in medicine, recreation, and industry, there is a dire need to apply new biotechnological tools to introduce new genotypes with desirable traits and enhanced secondary metabolite production. Micropropagation, conservation, cell suspension culture, hairy root culture, polyploidy manipulation, and *Agrobacterium*-mediated gene transformation have been studied and used in cannabis. However, some obstacles such as the low rate of transgenic plant regeneration and low efficiency of secondary metabolite production in hairy root culture and cell suspension culture have restricted the application of these approaches in cannabis. In the current review, in vitro culture and genetic engineering methods in cannabis along with other promising techniques such as morphogenic genes, new computational approaches, clustered regularly interspaced short palindromic repeats (CRISPR), CRISPR/Cas9-equipped *Agrobacterium*-mediated genome editing, and hairy root culture, that can help improve gene transformation and plant regeneration, as well as enhance secondary metabolite production, have been highlighted and discussed.

## 1. Introduction

*Cannabis sativa* L. is a high-demand plant with a long history of medicinal, industrial, recreational, and agricultural uses [1,2]. Cannabis can be categorized based on taxonomic relationships or chemotype but is often divided into two main groups and regulated based on the level of psychoactive cannabinoids that are produced. In most countries, anything below 0.3% Δ^9^-tetrahydrocannabinol (THC) is classified as hemp and plants that produce 0.3% or greater are categorized as marijuana [3]. To date, more than 560 secondary metabolites are known in cannabis [1,2]. Although cannabinoids and terpenes are the predominant biomolecules in cannabis, phenolic compounds and flavonoids have also been detected. Currently, more than 115 cannabinoids, isoprenylated polyketides, have been identified in cannabis, which are mainly produced in glandular trichomes of female flowers. Cannabidiol (CBD), THC, and cannabichromene (CBC) can be considered as the major cannabinoids in the crop, but new genetics that express other cannabinoids such as cannabigerol (CBG) are now emerging [4].

**Figure 1:**
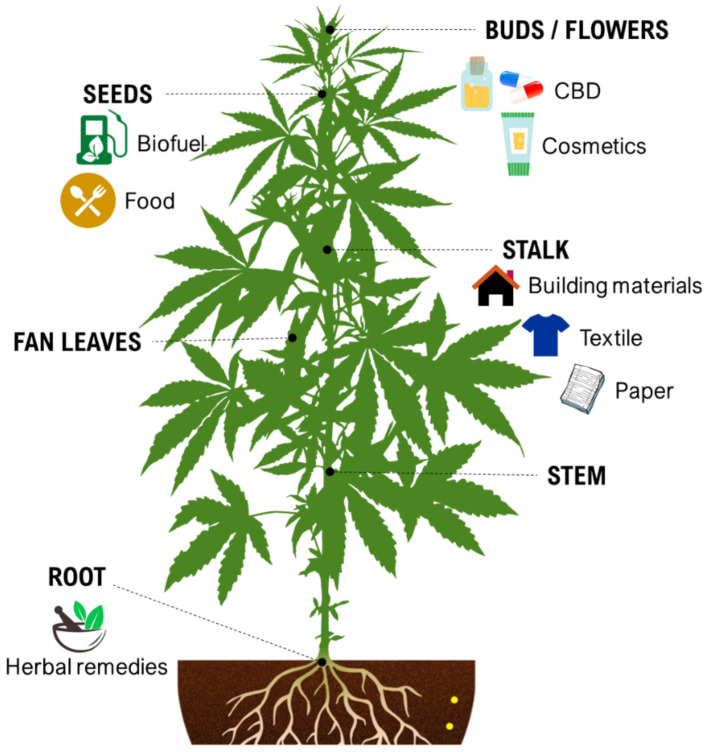
Some industrial properties of Cannabis.

During the last decade, the industrial properties of cannabis (Figure 1) for applications in textiles, paper, building materials, cosmetics, and foods [5,6,7], as well as pharmacological properties (Table 1) such as the palliation of chronic pains associated with cancer, neutralizing the adverse impacts of chemotherapy with cytostatic drugs, eating disorders related to anorexia and AIDS, inflammatory diseases, epilepsy, and anti-spastic activity in Tourette’s syndrome or sclerosis multiplex cases have been broadly studied and supported [5,8]. 

While cannabinoids and cannabinoid-containing products are a new market, they are exponentially growing and a recent market report estimated that the global value of CBD alone will reach 16 billion by 2025 [9]. As the demand for these products increases, there is a pressing need to develop improved genetics and cultivation techniques [10,11]. 

Conventional plant breeding involves directed crosses of parent plants with desirable characteristics, population evaluation, selection, and fixing desired traits (selfing). In cannabis, these are difficult criteria to meet due to plant biology (e.g., dioecy) and regulations. Cannabis plants are predominantly dioecious but selfing can be achieved through the induction of male flowers on female plants to produce feminized seeds [40]. These limitations in cannabis make conventional breeding methods time-consuming, costly, and laborious. The composition and content of cannabis secondary metabolites, in particular cannabinoids and terpenes, is also greatly related to various factors such as genotypes, age of plants, sex, developmental phase, growth and environmental conditions, harvesting time, storage conditions, and methods of cultivation [3,5]. 

For most crops with this economic importance, biotechnological tools (i.e., genetic engineering methods including transcription activator-like effector nucleases (TALENs) [41], zinc-finger nucleases (ZFNs) [42], and clustered regularly interspaced short palindromic repeats (CRISPR) [43] are well developed and have been implemented into breeding programs for decades. However, due to the long history of the prohibition of recreational/drug type cannabis, along with the strict regulation and lower market value of hemp, these tools are rudimentary, and many common techniques used in other crops have yet to be applied to cannabis [44]. With recent shifts towards the legalization of cannabis for medicinal and recreational purposes in many jurisdictions and the establishment of a legal market for cannabinoids, cannabis production is becoming a large-scale enterprise similar to other major crops [3]. Along with the emergence of legal commercial producers, the need for modern technologies for genetic improvement is steadily increasing. While biotechnology of cannabis is still relatively new and unrefined, with the advent of affordable large-scale sequencing technologies (i.e., next-generation sequencing (NGS)) and the increasing body of candidate genes for traits of interest, we argue that it is time for a paradigm shift toward improving cannabis genetics through genetic engineering.

Recently, whole genomic and transcriptomic information of cannabis has been obtained using NGS methods [1]. Cannabis NGS information can be applied for robust molecular tools such as DNA barcoding to detect genetic diversity, sex determination, and chemotype inheritance [5]. Moreover, these data can be merged with metabolomics and proteomics to identify unknown secondary metabolites of cannabis [3]. More rapid and accurate transcriptome analysis to detect key enzymes and genes in the biosynthetic pathway of secondary metabolites, mapping of unknown and wild populations using restriction site-associated DNA sequencing (e.g., genotyping by sequencing (GBS)) [45], and interpretation of targeting-induced local lesions in genomes (TILLING) populations are applicable based on the NGS information in cannabis [3,5]. Above all, NGS-derived data facilitate the introduction of genetic engineering methods in cannabis [46]. 

In vitro tissue culture techniques (e.g., callus and cell culture, de novo regeneration, hairy root culture) are the basis of micropropagation and breeding in cannabis [10,47]. In vitro culture methods coupled with genetic engineering techniques (e.g., *Agrobacterium*-mediated gene transformation and genome editing) as well as polyploidy induction offer opportunities for producing new genotypes and manipulating secondary metabolite production in cannabis [46]. Although conventional genetic engineering tools (e.g., *Agrobacterium*-mediated gene transformation and *A. rhizogenes*-mediated hairy root cultures) can alter the production of some secondary metabolites, it seems that the CRISPR/Cas9 system has more potential than these tools to introduce new germplasms and enhance secondary metabolite production in cannabis in a faster manner [10,47]. Therefore, biotechnological methods can be employed in order to develop improved genetics to help satisfy the demands of producers and consumers.

In the current review, all applied in vitro propagation and genetic engineering methods in cannabis along with other possibly applicable techniques such as designing new culture media, machine learning algorithms, and morphogenic genes that can help cannabis propagation and improvements, as well as enhance secondary metabolite yield, have been highlighted and discussed. The principles, benefits, weaknesses, and concerns of different methods have also been presented. 

## 2. In vitro Culture in Cannabis

In vitro culture is the basis of most biotechnological tools [10,47]. Many methods such as micropropagation, in situ and ex situ conservation, cell culture, *Agrobacterium*-mediated gene transformation, and polyploidy induction completely depend on in vitro culture techniques [48]. Moreover, plant cell and tissue culture is also a robust method for assessing the secondary metabolite production and endogenous phytohormone metabolism signaling in many plants [46]. Indeed, in vitro culture techniques are useful to propagate plants, but also to produce engineered biomolecules and initiate synthetic biology approaches [10,47].

Callus and cell suspension cultures were one of the main objectives of early in vitro culture in cannabis. The first attempts of callus cultures to produce cannabinoids were performed by Hemphill et al. [49], Loh et al. [50], and Braemer and Paris [51] and led to the conversion of olivetol and CBD to cannabielsoin. However, unstable and inadequate levels of cannabinoid production were achieved. Furthermore, cannabinoids could not be synthesized without adding exogenous cannabigerolic acid (CBGA) as a precursor to the callogenesis medium. Further studies [52,53] revealed that cannabinoids could not be produced even from an inflorescence-derived callus. In another study, Flores-Sanchez et al. [54] used various biotic (*Pythium aphanidermatum* and *Botrytis cinerea*) and abiotic (methyl jasmonate, salicylic acid, jasmonic acid, UV-B, AgNO_3_, NiSO_4_·6H_2_O, and CoCl_2_·6H_2_O) elicitors in cannabis cell suspension cultures; however, improved cannabinoid production was not obtained. 

These results suggest that the biosynthesis of cannabinoids is completely linked to tissue and organ-specific development and complex gene regulatory networks that can only be efficiently produced by trichomes, which are most abundant in differentiated floral tissues. However, cell suspension cultures may still be promising for producing other secondary metabolites such as terpenes, polyphenols, lignans, and alkaloids [7,10]. For instance, Gabotti et al. [55] reported that the activity and expression of tyrosine aminotransferase (TAT) and phenylalanine ammonia-lyase (PAL) increased in cannabis cell suspension cultures using a methyl jasmonate elicitor in combination with tyrosine precursor. Some aromatic compounds such as 4-hydroxyphenylpyruvate (4-HPP) were also identified. This is relevant as highly biologically active flavonoids have been isolated from cannabis [55].

Hairy root culture is another application of in vitro methods that have been used for secondary metabolite production in many species and investigated in cannabis [10]. Affordable and high production of secondary metabolites, high genetic stability, and rapid accumulation and growth of biomass are only some of the merits of hairy root cultures [46]. A larger scale and more profitable process can also be achieved by the cultivation of hairy roots in bioreactors [56]. Sirikantaramas et al. [57] isolated Δ^9^-tetrahydrocannabinolic acid synthase (THCAS) from cannabis leaves and cloned its cDNA. Then, the cDNA was transformed in tobacco hairy roots using *A. rhizogenes.* Although THCA was produced through THCAS expression and by adding CBGA, the THCA production rate was low. Farag and Kayser [58] reported 1 µg THCA g^−1^ dry weight (DW), 1.7 µg CBDA g^−1^ DW, 1.6 µg CBGA g^−1^ DW, and 2 µg cannabinoids g^−1^ DW obtained from adventitious roots from callus cultures. Given that floral tissues from whole plants can produce over 20% THC w/dw, these levels are very low [58]. These results are not surprising given that cannabinoids are generally produced in trichomes, which are not found in root tissues and this approach is likely not suitable for cannabinoids production. Generally, many compounds require differentiated tissues for efficient production. Moher et al. [59] demonstrated that in vitro plants respond to photoperiod and that they develop “normal” looking flowers. While the cannabinoid content of these flowers has not been examined, it is likely that they produce much higher levels than would be observed in undifferentiated tissues, or roots. Therefore, this could be an alternative approach to producing cannabinoids in vitro but it has yet to be explored.

**Figure 2:**
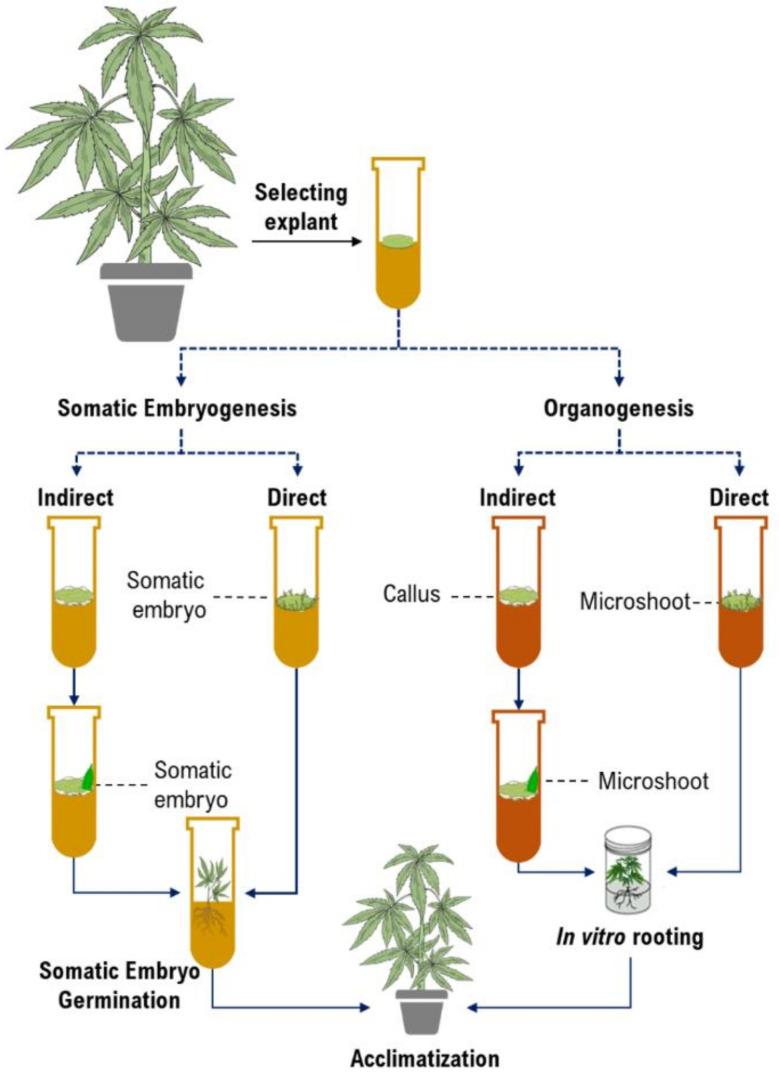
The schematic diagram of plant tissue culture procedures.

Micropropagation is the first and foremost application of in vitro culture in cannabis [10,47,60,61]. While micropropagation for applications in genetic preservation or propagation are generally achieved through shoot proliferation from existing meristems, many applications in biotechnology require the establishment of de novo regeneration in which plants are produced from non-meristematic tissues. Somatic embryogenesis and organogenesis through either direct or indirect regeneration are the most important platforms for developing regeneration protocols (Figure 2). Although somatic embryogenesis is considered the ideal approach since they regenerate from single cells and reduce chimerism in transformed plants [46], it has been rarely achieved in cannabis. Table 2 represents callogenesis and organogenesis studies in cannabis to date. As can be seen in Table 2, most studies have investigated the effects of plant growth regulators (PGRs) and type of explants and genotypes on micropropagation of cannabis. However, there are many factors (e.g., medium composition and incubation conditions, discussed in the following sections) that affect cannabis micropropagation. Therefore, it is necessary to study these factors for obtaining high-frequency protocols.

### 2.1. Strategies to Improve In Vitro Culture Procedures

**Figure 3:**
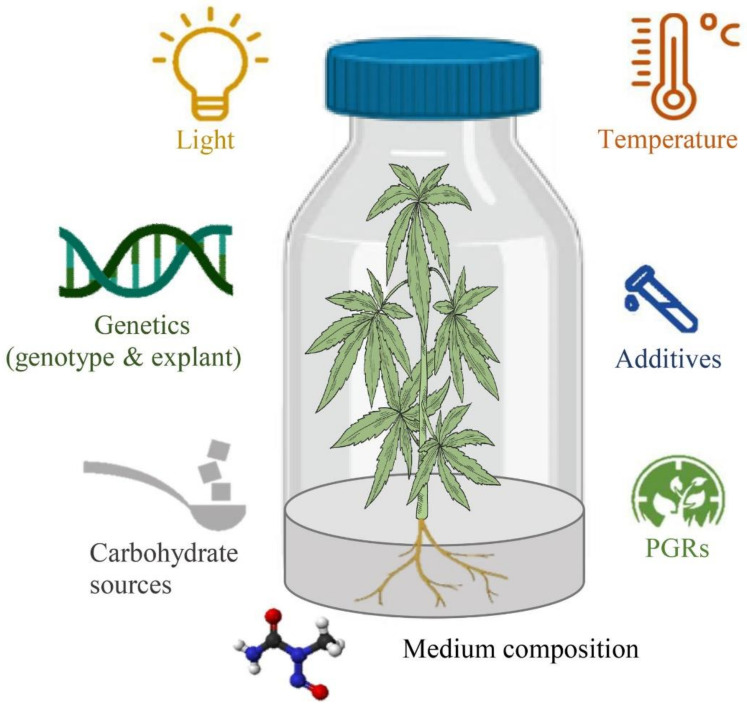
The schematic diagram of factors affecting in vitro culture procedures.

Despite advances in in vitro cell and tissue culture of cannabis in recent years, efficient cannabis regeneration remains one of the main obstacles to applying biotechnology for cannabis improvement and the species is generally considered to be relatively recalcitrant [94]. Genotypes, type and concentration of PGRs, size, age, and type of explant, gelling agent, carbohydrate sources, type and concentration of macro- and micro-nutrients, type and concentration of vitamins, type and concentration of additives (casein hydrolysate, nanoparticles, phloroglucinol, activated charcoal, etc.), pH of the medium, type and volume of the vessels, volume of the medium per culture vessels, and culture conditions (intensity and quality of the light, temperature, photoperiod, and light source) are the most important factors affecting in vitro culture systems [94,95] (Figure 3). However, most studies have investigated the effects of PGRs and the type of explants and genotypes on cannabis micropropagation and little information on many other factors is available. Therefore, studying other factors may result in high-frequency regeneration systems or even obtaining somatic embryogenesis and haploid production protocols. In this section, several promising strategies for improving in vitro culture protocols have been highlighted based on the mentioned factors, new computational methodologies such as machine learning algorithms, and new genetic engineering methods.

Although a few studies [78,81] have tested newer PGRs and additives such as 6-benzylamino-9-(tetrahydroxypyranyl) purin (BAP9THP) and meta-topolin (mT) for cannabis micropropagation, there are still some promising PGRs and additives, such as polyamines, brassinosteroids, nano-particles, and nitric oxide (NO) that have not been used for developing cannabis micropropagation protocols. NO, a messenger molecule regulating plant development such as flowering, germination, fruit ripening, and organ senescence, has been recently characterized as a phytohormone [96,97]. It was shown that NO can be experimentally applied in the media as sodium nitroprusside (SNP), which eliminates the difficulty in the application of NO in its gaseous form [98]. Several studies showed that NO is one of the main signaling pathways in in vitro organogenesis and somatic embryogenesis [96,99]. Therefore, the application of SNP may pave the way for obtaining somatic embryogenesis or improving organogenesis protocols in cannabis. Recent studies showed that adding nanoparticles to the culture media improves callogenesis, organogenesis, somatic embryogenesis, and rhizogenesis by inhibiting the production of ROS and ethylene and altering gene expression and antioxidant enzyme activities [100,101,102,103,104,105]. Thus, the application of nanoparticles can be investigated as a promising approach to enhance the in vitro regeneration capacity of cannabis.

The source of carbohydrates is another factor affecting in vitro culture systems. The effect of sucrose, glucose, and fructose as the most important carbohydrates have been widely studied in in vitro morphogenic responses of different plants [106]. While sucrose has resulted in the maximum in vitro organogenesis and embryogenesis in some plants (e.g., *Agave angustifolia* [107], *Sapindus trifoliatus* [108], and *Pinus koraiensis* [109]), other plants (e.g., *Vitis Vinifera* [110], *Brassica napus* [111], and *Chrysanthemum ×grandiflorum* [112]) had better in vitro morphogenic responses to glucose and fructose [106]. Therefore, it is essential to study the effect of different carbohydrate sources on cannabis micropropagation.

The source, intensity, and quality of light play a pivotal role in *in vitro* organogenesis and embryogenesis [112,113]. The usefulness of light emitting diodes (LEDs) in different micropropagation procedures has been widely demonstrated [114,115]. LEDs provide an appropriate light spectrum and therefore can be considered promising light sources for improving micropropagation studies [113,116]. However, it has been shown that each step of in vitro culture needs a particular light spectrum [113]. For instance, there is an ongoing debate on using red or blue light. However, several studies showed that the red light is better than blue light for somatic embryogenesis [112,117].

Many cannabis micropropagation studies [65,66,67,69,71,72,73,74,75,76,77,78,79,80,81,83] used MS [118] as a basal medium while the composition of MS medium was initially defined for the analysis of tissue ashes of tobacco. Several factors related to the basal medium, such as macro- and micro-elements and vitamins, are known as major factors that are affected in vitro morphogenesis in different species or plant organs [119]. Recently, Page et al. [92] reported that plants cultivated on MS medium displayed a number of physiological defects and that DKW [120] basal salts were much better. Additionally, they reported that DKW basal salts also supported greater callus growth from leaf explants. Together, this suggests that MS salts are sub-optimal for shoot and callus growth in cannabis, but the authors also stated that plants cultured on DKW basal salts still displayed some symptoms and further improvement is likely possible and they did not report regeneration so it is unknown if DKW is suitable for that application. 

The challenges in designing a de novo medium and optimizing these myriad factors for specific purposes are expensive and time consuming due to the large number of variables and their interactions with one another. Therefore, new approaches such as new computational methodologies (i.e., machine learning algorithms) are needed to design regeneration protocols. Artificial intelligence models and optimization algorithms provide a complementary outlook for calibrating in vitro protocols, as these algorithms find optimal solutions in terms of genotype, explant source, plant growth regulators, medium composition, and incubation conditions, without the requirement for large-scale, costly, time-consuming, and tedious experimental trials [95,121]. Recently, different machine learning algorithms have been successfully used for predicting and optimizing different in vitro culture processes such as shoot proliferation [122,123,124,125,126], callogenesis [127,128], somatic embryogenesis [129], secondary metabolite production [130,131,132], and gene transformation [133]. Hence, the combination of the experimental approach and machine learning algorithms can be considered a powerful and reliable method to develop a specific protocol for cannabis.

It has been shown that micropropagation after mechanical wounding induced by brushing tissue surfaces has been significantly increased [134]. Although there are no reports regarding the effect of wounding on cannabis micropropagation, from our observations callusing has generally initialed at wound sites and tissue wounding may be a promising approach to improve plant regeneration in cannabis. Three consecutive stages improve in vitro organogenesis by tissue wounding: (i) organogenesis is stimulated by some signals related to tissue damages, (ii) subsequently, endogenous phytohormones are accumulated, which results in (iii) cell fate transition [134]. 

Thin cell layer culture can be considered as another promising approach that can be used in cannabis micropropagation [135]. Although this method has been used in different recalcitrant plants such as *Hedychium coronarium* [136], *Withania coagulans* [135], and *Agave fourcroydes* [137], there is no report of the application of thin cell layer culture in cannabis. In this method, a thin layer of tissue as the explant is selected, which causes close contact between wounded cells and medium composition and finally leads to improvement of regeneration [135].

Bioreactors (e.g., continuous immersion and temporary immersion) can be considered as useful tools for cannabis micropropagation and for studying plant development [138]. The use of these devices can help overcome the recalcitrance of cannabis genotypes to proliferation, rooting, and acclimation. In addition, they can also be used to reduce the cost of large-scale propagation. The number of cannabis plants cultured in bioreactors is steadily increased, and frequently the physiological state of plant propagules improves with these systems of culture, which also facilitate photoautotrophic propagation.

The use of morphogenic genes is another strategy that may help alleviate the bottlenecks in cannabis regeneration. This strategy has been extensively discussed in section “4.2. Strategies to Improve Gene Transformation Efficiency”.

Protoplast culture can be considered a powerful method for many purposes such as plant regeneration, functional genetic analyses, genome editing, and studying cell processes (e.g., membrane function, cell structure, and hormonal signalization) [139,140]. The development of reproducible and stable protoplast isolation is one of the most important prerequisites for the success of protoplast-based technology. Although there are a few studies about protoplast isolation in cannabis (Table 3), there is no report regarding protoplast-mediated plant regeneration. 

Beard et al. [141] showed protoplast isolation from the mesophyll of young, not fully expanded leaves of in vitro grown plantlets of *C. sativa* var. Cherry x Otto II: Sweetened. The authors reported that an enzymolysis solution composed of 0.3% w/v Macerozyme R-10, 20 mM MES (2-(N-morpholino) ethanesulfonic acid), 1.25% w/v Cellulase R-10, 0.4 M mannitol, 0.1% w/v bovine serum albumin, 10 mM calcium chloride, 20 mM potassium chloride, and 0.075% w/v Pectolyase Y23, adjusted to pH 5.7 and heated to 55 °C for 10 min, resulted in the maximum number of protoplasts (2.27 × 10^6^ protoplasts per gram of leaf segments). Lazič [142] showed protoplast isolation from etiolated hypocotyls and the mesophyll of leaf cells of cannabis. The author also reported that an enzyme solution composed of 0.4% Macerozyme R–10 and 1.5% Cellulase Onozuka R-10 resulted in the maximum number of protoplasts from leaves whereas the highest number of protoplasts from etiolated hypocotyls was achieved from enzyme solution supplemented with 0.1% Macerozyme R-10 and 1% Cellulase Onozuka R-10. In another study, cannabis protoplasts were isolated using a digestion solution supplemented with 88 mM sucrose, 0.4 M mannitol, 1% (w/v) Cellulase Onozuka R-10, 0.1% (w/v) pectolyase Y-23, and 0.2% (w/v) Macerozyme R-10 at 30 °C for 4 h with gentle agitation [143].

Cannabis is a dioecious species, with separate male and female plants, and the most economically important product is unfertilized, seedless, female flowers [1]. Some of the challenges that result from these factors include producers not being able to have pollen-producing plants in their production facility, plants must be unfertilized for accurate phenotyping, which complicates breeding strategies, and it is difficult to self-pollenate plants to produce inbred lines for F1 hybrid seed production [48]. To address these challenges, in vitro techniques for the production of homozygous double haploids for F1 hybrid production can be considered as a robust solution [128]. In vitro haploid production consists of different methods such as wide hybridization-chromosome elimination, parthenogenesis, gynogenesis, and androgenesis [144]. Although there are no reports regarding haploid production in cannabis, it seems that haploid production protocols are needed for further genetic engineering studies. Recently, knockdown and/or knockout of the centromere-specific histone H3 (CENH3) gene, which connects spindle microtubules to chromosome centromere regions, provides a robust tool for producing haploid plants [145,146]. For instance, Wang et al. [145] and Kelliher et al. [146] successfully used the CRISPR/Cas9 system for the knockout of the CENH3 gene in maize genotypes to produce haploid inducer lines. It seems that such methodologies are very useful for haploid production in cannabis [147]; however, it is vital to develop stable gene transformation and plant regeneration systems before this can be done.

A combination of polyploidy induction and CRISPR/Cas9-equipped *Agrobacterium rhizogenes*-mediated hairy root culture can be considered as a robust strategy for increasing secondary metabolites production and changing the chemical profile [46]. This strategy has been recently applied for the knockout of the *Sm*CPS1, an important gene in the tanshinone biosynthesis pathway [148] and *Sm*RAS, a key gene in rosmarinic acid biosynthesis, [149] in *Salvia miltiorrhiza*, as well as *Dz*FPS, a key gene in farnesyl pyrophosphate biosynthesis, in *Dioscorea zingiberensis* [150]. It seems that this strategy can be used to overcome the problems in hairy root culture of cannabis.

### 2.2. Somaclonal Variation

**Figure 4:**
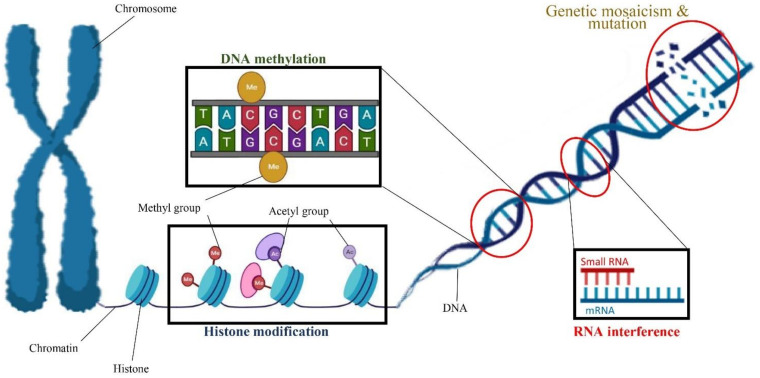
A schematic view of factors involved in somaclonal variation including genetic mosaicism and mutation as well as epigenetic regulations such as DNA methylation, histone modification, and RNA interference.

Most previous cannabis tissue culture studies have focused on optimizing culture conditions to increase the cannabis micropropagation rate. However, the optimal condition for in vitro propagation may not be optimal to preserve the genetic integrity of the regenerated genotype [151]. Indeed, in vitro conditions such as medium composition, PGRs, high humidity, the number of subcultures, length of the culture period, temperature, light quality, and light intensity can eventually result in several developmental and physiological aberrations of the micropropagated plants [60]. The term “somaclonal variation” refers to any phenotypic variation detected among micropropagated plants [151]. Somaclonal variation is created by either chromosome mosaics and spontaneous mutation or epigenetic regulations such as histone modification (e.g., histone methylation and histone acetylation), DNA methylation, and RNA interference [151,152] (Figure 4). 

Somaclonal variation can be considered as a double-edged sword that has its own merits and demerits based on the objective of the micropropagation experiment. If the objective of micropropagation is breeding, increasing diversity, and generating new variants, somaclonal variation can be considered a beneficial event. On the other hand, if the objective of micropropagation is the production of true-to-type clones, somaclonal variation can be considered as an obstacle.

Although previous cannabis tissue culture studies have shown that regenerated cannabis plants are phenotypically similar to the mother plants and genetically stable with a low mutation rate [78,81,153,154,155], they employed low-resolution molecular markers such as Inter Simple Sequence Repeats (ISSR), which leads to the detection of somaclonal variation, only, at specific genomic regions. Recently, Adamek et al. [156] employed deep whole-genome sequencing to determine the accumulation of somatic mutations within different parts of an individual *Cannabis sativa* cv. “Honey Banana” plant. They identified a significant number of intra-plant genetic diversity that could impact the long-term genetic fidelity of clonal lines and potentially contribute to the phenotypic variation. Application of the new approaches based on NGS technologies in combination with epigenetic studies is required for the future investigation of the mutation rate in micropropagated cannabis.

## 3. Ploidy Engineering in Cannabis

Polyploidy is common in many cultivated crop species including wheat, banana, potato, sugar cane, rye, alfalfa, apple, and strawberry [157]; however, the way in which each crop harnesses the benefits of polyploidy is unique. Polyploid can be used as a method of increasing heterosis in a population or can help to mask deleterious alleles [158]. The ploidy level of crops can also be manipulated to induce desired characteristics such as seedless fruits and is achieved by crossing two individuals with unique ploidy levels to produce progeny with an odd number of chromosomes [157]. This is a desired characteristic in cannabis production as seedless flowers produce a greater economic yield [48]. As the production of cannabis moves outdoors, seedless cultivars will likely become increasingly popular. Cannabis is naturally a diploid and multiple successful artificial inductions of polyploidy in cannabis have been reported [159,160,161]. Kurtz et al. [160] have used this technique to produce triploid plants, but field performance and lack of seed development have not yet been reported. These reports provide promising results for the potential for polyploidy to be used to improve cannabis cultivars. Polyploidy induction studies in cannabis have been summarized in Table 4.

### 3.1. Types of Polyploids

Polyploidy occurs when the normal somatic cells of an organism have more than two sets of homologous chromosomes [158]. An organism can also be a chimera where the individual is composed of cells with different numbers of chromosomes. If the DNA content within the chimera has various ploidy levels, then the organism is a mixoploid [157]. Autopolyploid is defined as polyploidization within a single species and can be produced either somatically or sexually [158]. 

Somatic polyploids are produced using anti-mitotic agents that are intended to alter the process of mitosis inducing irregular cell division [157]. Oryzalin has been proven to effectively disrupt the action of mitosis in plants through disruption of microtubule action [158]. In contrast, allopolyploid is described as a polyploidization as a result of a hybridization between two unique species, which has not yet been documented for cannabis [158,161]. 

### 3.2. Advantages to Polyploidy in Breeding Programs

The natural production of polyploids is considered to be one of the major mechanisms of speciation [158]. The formation of polyploids in nature creates increased heterosis, which could potentially be exploited by modern breeders.

A major benefit of polyploidy is the ability to produce seedless triploids [157]. The production of seedless plants requires crossing two individuals with different ploidy levels. This is usually done by crossing a tetraploid and diploid plant [158]. As both the diploid and tetraploid organisms contain even sets of chromosomes the pairs segregate normally [163]. The two gametes fuse in the mother and produce a triploid (2n = 3x) embryo [163]. The triploid embryo is viable and can undergo regular cell division. The seedless mechanism in triploids alters meiosis so that viable gametes are not produced. Due to the inability of the triploid plant to produce viable gametes, seed production is aborted [164]. Seedless cultivars of cannabis are particularly valuable as studies have shown that seed sets reduce the production of secondary metabolites [165]. This is of particular interest to commercial operations as production moves outdoor. 

### 3.3. Disadvantages to Polyploid Breeding

While autopolyploids provide many benefits to breeders, there are also some fundamental problems with breeding at higher ploidy levels. One drawback to polyploid breeding is that heterozygotes and homozygotes do not separate into classic mendelian ratios [157]. This becomes a significant issue when selecting for disease resistance or selecting more than one recessive trait [158]. Another issue breeders face is that combining two recessive traits becomes more difficult where the chance of getting a double recessive genotype decreases from 1/16 to 1/1296 in tetraploids [157]. This suggests that if the goal of a polyploid breeding program is to combine two or more recessive alleles it would be beneficial to make these improvements at the diploid level before polyploidization. Finally, severe inbreeding depression in polyploid cannabis could render this mechanism useless [163].

### 3.4. Effects of Polyploidy

**Figure 5:**
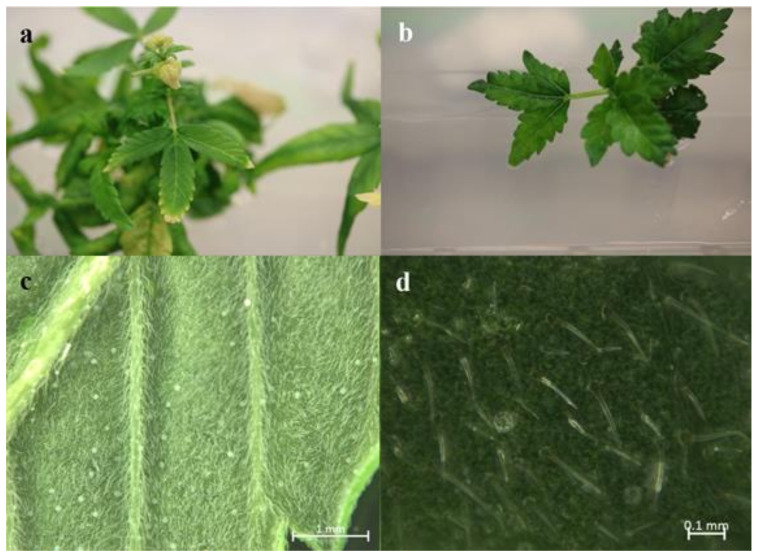
Comparing the morphological traits of diploid and tetraploid cannabis (**a**) Diploid Cannabis leaf, (**b**) Tetraploid Cannabis leaf, which is noticeably wider than the diploid, (**c**) Bright light image of diploid Cannabis stomata, (**d**) Bright light image of tetraploid Cannabis stomata.

Studies observing the morphological traits of tetraploid hemp-type cannabis have shown differences in leaf width, stomate count, and stomate size compared to diploid plants (Figure 5) [159]. The tetraploid leaves were 47% larger than diploid leaves and the tetraploid flowers were more than twice the diameter in comparison to the control flowers [159]. Stomates in the tetraploid plants were twice the length of the diploid stomates but the stomate density was lower [159]. The above-ground shoot weight of the tetraploid plants was almost twice the mass of the diploids. At the cellular level, it is noted that tetraploid individuals have larger mesophyll cells and less intercellular space [162].

A recent study [161] reported a successful in vitro polyploidy induction in a drug type cannabis using oryzalin. In this study, growth media was supplemented with various concentrations of oryzalin ranging from 20–150 µM and clonal explants from a greenhouse were exposed to treatments for 24 h. The most successful treatments reported were 20 and 40 µM concentrations [161]. Two cultivars were used in this trial and results did vary between treatments. For one cultivar, the 20 µM treatment was sufficient to induce polyploidy; however, for the second cultivar the 20 µM concentration did not produce tetraploids and the 40 µM treatment was most successful. It was noted that following treatments it took several weeks for explants to show any signs of growth. Following treatments, explants were acclimatized and transferred to a greenhouse to observe growth. Tetraploid plants showed an increase in rooting time and a decrease in rooting success compared to diploids [161]. The polyploid plants had slight morphological differences compared to diploids. Tetraploid plants had wider leaves, larger stomates, and a lower density of stomates compared to diploids [161]. The effect of polyploidy on phytochemical composition was noted and CBDA was the only cannabinoid that increased in the polyploid population. In addition, the terpene content of the cannabis plants was also increased in the polyploid population [161].

### 3.5. Secondary Metabolites

Many species that produce secondary metabolites have witnessed an increased production of these compounds at higher ploidy levels [158]. An increase of secondary metabolite production yield has been reported in polyploid *Vetiveria zizanioides* L. Nash compared to its diploid counterpart [166]. This species produces aromatic compounds valuable to the fragrance industry and production of these compounds was increased by over 62% when polyploidy was induced [166]. As secondary metabolites produced by cannabis are becoming a legal commodity, the production of these compounds needs to be optimized. In recent literature, it has been reported that polyploid cannabis plants had lower THC production compared to diploid controls but also had increased CBD production [161]. In hemp, the polyploid individuals produced on average 50% less THC in the female flowers compared to the control, but CBD production in the female leaves was more than three times greater in the polyploid population [162]. This study utilized a hemp variety of cannabis, which is bred for low secondary metabolite production [162]. Duplication of some deleterious recessive alleles may be responsible for the decreased THC concentration observed in polyploid cannabis plants. A deleterious allele affecting an important enzyme involved in the metabolic pathway of cannabinoid synthesis could inhibit the entire process. Secondary metabolites such as cannabinoids are heavily dependent on the presence of chemical precursors and enzymes [158].

### 3.6. Limitations of Existing Polyploidy Literature and Future Potential

Based on the current literature there is very little reported work in the interest of polyploidization in cannabis, with only moderate morphological/chemical differences [161,162]. However, it should be noted that the existing literature does not evaluate many agronomically important traits and only evaluated the first generation of artificially induced autotetraploids. It is worthwhile to mention that, while the tetraploids hold twice as many chromosomes, they do not contain a greater number of unique alleles. Further studies including crosses of unique tetraploids are needed to fully understand the effects of tetraploidy that includes greater allelic diversity. It is possible that while the initial generation of tetraploids is similar to their diploid progenitors, subsequent generations may demonstrate unique phenotypes that are of use to modern breeding programs. Regardless, the use of tetraploids to produce seedless triploids has great potential for the cannabis industry. Moreover, ploidy engineering can be used in cannabis for terpene manipulation, CBD-to-THC ratio in hemp, biomass improvements, and novel cannabinoid production.

## 4. Genetic Engineering Approaches in Cannabis

Plant genetic engineering can be considered a basic approach to studying gene function and genetic improvement. Generally, plant cells can be either transiently or stably transformed [167]. Although there are a few studies [168,169] that used targeting-induced local lesions in genomes (TILLING) and virus-induced gene silencing (VIGS) approaches for studying the function of some genes in cannabis, there is still a dire need for developing a stable gene transformation system [93]. TILLING, as a powerful method for selecting mutations in specific genes, was used by Bielecka et al. [168] to find cannabis plants with mutations in *CsFAD2* and *CsFAD3* genes that result in the modification of the seed-oil composition. The requirement of large mutant populations and homozygous mutations are the flip side of the TILLING method. Recently, the VIGS system using Cotton leaf crumple virus (CLCrV) was successfully applied in cannabis to knockdown endogenous phytoene desaturase (PDS) and magnesium chelatase subunit I (ChlI) genes [169]. 

**Figure 6:**
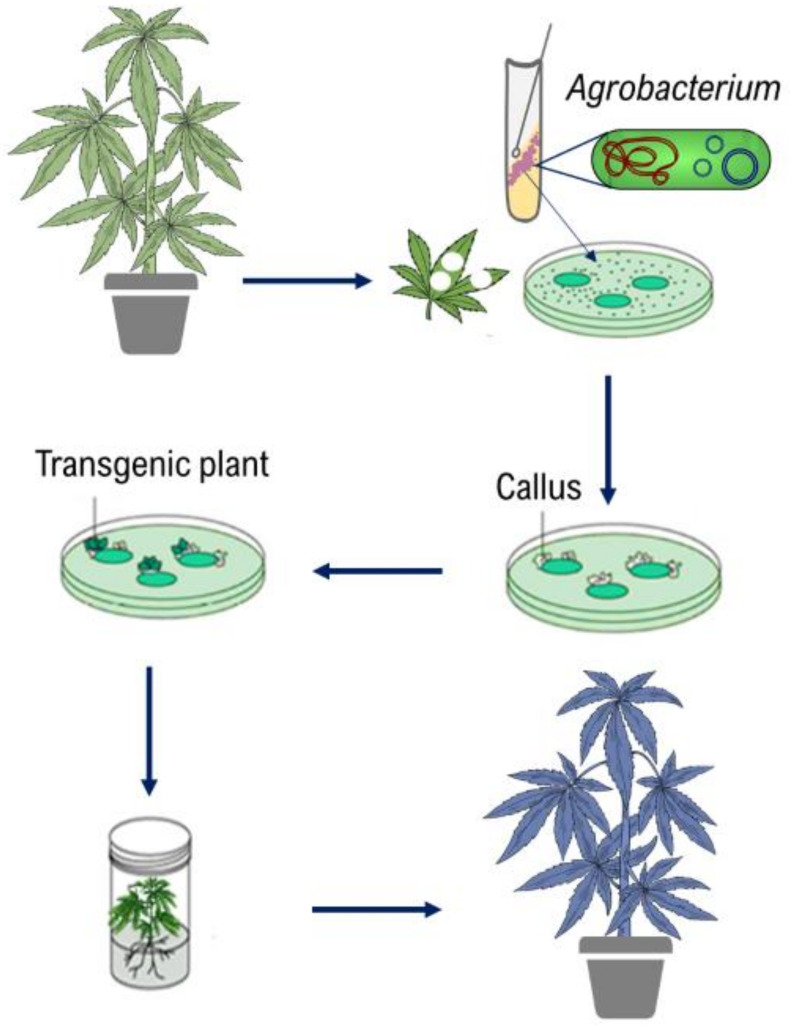
The schematic diagram of *Agrobacterium*-mediated gene transformation.

Genetic transformation allows foreign genes to be introduced into a crop and has been extensively used to introduce a variety of important traits (herbicide resistance, pro-vitamin A production, insect resistance, etc.) into major crops for decades [167]. CRISPR/Cas systems have also been recently applied for modifying major crops such as wheat and rice [170]. Developing a gene transformation and/or genome editing systems in cannabis are not only useful for modifying horticultural traits, growth morphology, and biotic and abiotic stress resistance but are also important for studying gene functions. *Agrobacterium*- and Biolistic-mediated gene transformation systems, de novo meristem induction, and virus-assisted gene editing are applicable to cannabis. Biolistic-mediated gene transformation, which uses particle bombardment to transfer the gene into the plant, and genome editing methods have not yet been reported in cannabis. On the other hand, several studies have investigated *Agrobacterium*-mediated gene transformation in cannabis. The *Agrobacterium*-mediated gene transformation system is directly dependent on plant tissue culture (Figure 6).

Recently, Beard et al. [141] used the protoplast of *C. sativa* var. Cherry x Otto II: Sweetened for transient transformation with plasmid DNA containing a fluorescent marker gene. The authors reported that more than 31% of the cells were successfully transformed. Although gene transformation has been achieved in cannabis by different studies [141,171,172,173,174,175,176], there is only one report regarding transgenic plant regeneration [93]. 

In the following section, cannabis transformation studies, factors involved in gene transformation, and strategies for improving gene transformation have been discussed.

### 4.1. Agrobacterium-Mediated Gene Transformation 

As soon as the susceptibility of cannabis genotypes to *Agrobacterium* was revealed [171], *Agrobacterium*-mediated gene transformation in cannabis became of great interest to many. However, several obstacles have been reported for establishing and developing gene transformation in cannabis such as low efficiency of gene transformation, low rates of regeneration, chimeric regeneration including both non-transgenic and transgenic cells and tissues, as well as inactivation of the transgene [10,47]. Therefore, it is crucial to study different factors involved in gene transformation such as *Agrobacterium* strains, treatments for explants infection, selection markers, eliminating chimerism, promoters, and translational enhancer. *Agrobacterium*-mediated gene transformation studies have been summarized in Table 5.

#### 4.1.1. *Agrobacterium* Strains

*Agrobacterium* strain selection is one of the most important factors in gene transformation (Table 5). The first study of successful gene transformation with more than 50% transformation frequency in fiber-type cannabis (hemp) was performed by MacKinnon et al. [171]. However, they did not report which strain of *Agrobacterium* was used. Feeney and Punja [172] obtained an acceptable transformation efficiency (15.1 to 55.3%) by using *A. tumefaciens* EHA101. Wahby et al. [173] used three *A. tumefaciens* strains including LBA4404, C58, and IVIA 251, as well as eight *A. rhizogenes* strains including 476, 477, 478, A424, AR10GUS, A4, AR10, and R1601 for establishing hairy root cultures in different genotypes of cannabis. According to their results, genotypes had different responses to *Agrobacterium* strains in such a way that transformation efficiency ranged between 43% for AR10GUS to 98% for R1601 in *A. rhizogenes* strains, and between 33.7% for IVIA251 and 63% for C58 for *A. tumefaciens* strains. Generally, Wahby et al. [173] reported that the gene transformation frequency in cannabis is dependent not only on the strains of *Agrobacterium* but also on cannabis genotypes, consisting of their sensitivity to agro-infection and their potential to regenerate transgenic tissues.

Deguchi et al. [175] compared transformation efficiency among several hemp genotypes in including Ferimon, Fedora 17, USO31, Felina 32, Santhica 27, Futura 75, CRS-1, and CFX-2 using different *A. tumefaciens* strains including LBA4404, GV3101, and EHA105 and found high transformation efficiency (>50%) for some genotypes. Based on their results, the maximum GUS expression was observed in the CRS-1 genotype and *A. tumefaciens* GV3101 led to the highest transformation frequency. In another study, Sorokin et al. [176] investigated the potential of *A. tumefaciens* EHA105 in transforming different cannabis genotypes (Candida CD-1, Holy Grail x CD-1, Green Crack CBD, and Nightingale) and obtained a high transformation efficiency (45–70.6%). Different responses to *Agrobacterium* strains are not unique to cannabis and it has been previously documented that various *Agrobacterium* strains differ in their capacity to transform different recalcitrant plants such as maize [177]. Therefore, it is necessary to investigate more strains to obtain efficient strains for a high-frequency gene transformation protocol.

#### 4.1.2. Infection of Explant

The physiological condition and source of explants play a pivotal role in *Agrobacterium*-mediated gene transformation. Different explants such as shoot tip and hypocotyl have been employed for gene transformation in cannabis (Table 5). Most studies used different parts of in vitro grown seedlings. MacKinnon et al. [171] succeeded in gene transformation using shoot tip explants that were selected from greenhouse-grown cannabis. Feeney and Punja [172] used callus cells derived from stem and leaf segments of cannabis for *Agrobacterium*-mediated gene transformation. In another study, Wahby et al. [173] used different parts of 5-day-old in vitro grown seedling of hemp including hypocotyls, cotyledons, cotyledonary node, and primary leaves for gene transformation and reported that the best gene transformation results were obtained from hypocotyl segments. Sorokin et al. [176] also used cotyledons and true leaves of 4-day-old in vitro grown seedling of hemp for gene transformation. Deguchi et al. [175] reported a successful gene transformation using male and female flowers, stem, leaf, and root tissues derived from 2-month-old in vitro grown seedling of hemp.

The co-cultivation period and concentration of *Agrobacterium* inoculum (optical density (OD)) have a significant impact on successful gene transformation. Feeney and Punja [172] suggested three-day co-cultivation and OD_600nm_ 1.6–1.8 for gene transformation of callus cells. In another study, different explants were co-cultured for two days [173]. Sorokin et al. [176] suggested three days of co-cultivation and OD_600nm_ 0.6 for gene transformation of different parts of in vitro grown seedling of hemp.

*Agrobacterium* infection efficiency in cannabis can be increased by adding chemical compounds, such as sodium citrate, acetosyringone, and mannose, to the co-cultivation medium. Feeney and Punja [172] reported increased *Agrobacterium* infection using 100 µM acetosyringone and 2% mannose for hemp gene transformation in the co-cultivation medium. Wahby et al. [173] studied the effect of different concentrations of acetosyringone (20, 100, and 200 µM), sucrose (0.5 and 2%), sodium citrate (20 mM), and 2-N-morpholineethanesulfonic acid (MES) (30 mM) on the gene transformation of cannabis and reported that different chemical compounds had little impact on *Agrobacterium* infection efficiency, and 20 µM acetosyringone resulted in the best results. On the other hand, Deguchi et al. [175] applied 200 μM acetosyringone, 2% glucose, and 10 mM MES for increasing strain virulence in the gene transformation of hemp. Sorokin et al. [176] also reported increasing the *Agrobacterium* infectability by using 100 µM acetosyringone for cannabis gene transformation in the co-cultivation medium. Recently, Karthik et al. [178] reported that SNP improved the efficiency of *Agrobacterium*-mediated gene transformation in soybean. Therefore, it is reasonable to investigate the effects of SNP on the gene transformation of cannabis. 

#### 4.1.3. Selection Markers

Although kanamycin has been the main selection agent of transgenic cannabis cells and tissues, other antibiotics, such as spectinomycin, rifampicin, and chloramphenicol, have also been successfully applied for selecting the transformed cells and tissues of cannabis [10,47]. However, it is necessary to study the effect of other antibiotics on cannabis gene transformation because the response of various tissues and genotypes to different antibiotics may vary. For instance, Sorokin et al. [176] and Feeney and Punja [174] used spectinomycin- and kanamycin-resistant genes as selectable markers in the *Agrobacterium* vectors. Wahby et al. [173] used *Agrobacterium* vectors carrying kanamycin-, carbenicillin-, and rifampicin-resistant genes for transforming cannabis. Sorokin et al. [176] also used kanamycin- and rifampicin-resistant genes in *Agrobacterium* vectors. Moreover, Deguchi et al. [175] considered the chloramphenicol-resistant gene as a selective marker in *Agrobacterium* vectors. 

Twin T-DNA binary vectors have also been successfully used for generating marker-free transgenic plants. This would be a very useful and promising method for generating marker-free transgenic cannabis and to mitigate scientific and public concerns regarding dispersing herbicide- and antibiotic-resistant genes of GMO products into the environment.

#### 4.1.4. Eliminating Chimerism

The regeneration of chimeric tissue with both non-transformed and transformed cells and tissues is one of the most crucial challenges in developing a stable gene transformation system in different plants [179]. Therefore, it is essential to use an approach to eliminate the chimeric cells and regenerate only transgenic cells. Feeney and Punja [172] studied the gene transformation frequency and chimerism using the phosphomannose isomerase (PMI) selection strategy, which is based on the existence of sugar (mannose) in the medium. They compared two transformation procedures including 1% mannose and 300 mg/L Timentin (treatment 1) and 2% mannose and 150 mg/L Timentin (treatment 2) and reported that treatment 1 was not capable of distinguishing non-transgenic cells from transgenic cells, and, therefore, they suggested treatment 2 for gene transformation in cannabis. Wahby et al. [173] compared the transformation performance and chimerism of two procedures of gene transformation: complex media MI1 (100 µM acetosyringone, 0.5% sucrose, and 30 mM MES) and MI2 (200 µM acetosyringone, 2% sucrose, 20 mM sodium citrate) and reported that these media could not completely detect transgenic tissues from non-transgenic tissues. Chimerism in transformation systems is not unique to cannabis and is a challenge in many species and is highly dependent on the regeneration system. Moving forward, developing an efficient somatic embryogenesis-based regeneration system will be important to mitigate this issue.

#### 4.1.5. Promoters and Translational Enhancer

Sorokin et al. [176] and Feeney and Punja [174] used the binary vector pNOV3635 and pCAMBIA1301, respectively, carrying a coding region for PMI under control of the nopaline synthase terminator (NOS) and the ubiquitin promoter derived from *Arabidopsis thaliana* (Ubq3), as well as a spectinomycin and kanamycin selectable marker genes. They also used chlorophenol-red PMI assay, PCR, and southern blot for confirming gene transformation.

When the β-glucuronidase (GUS) reporter gene was employed using the cauliflower mosaic virus 35S RNA (CaMV35S) promoter, GUS activities were observed [173]. In another study, Deguchi et al. [175] used a pEarleyGate 101 vector harboring the eGFP gene and uidA gene under the control of the CaMV 35 S promoter and OCS terminator for GFP fluorescence and GUS staining assays. Moreover, Sorokin et al. [176] reported that when the GUS gene in the pCAMBIA1301 vector was under the control of a CaMV 35S:: Intron, the highest GUS activity in the transgenic cannabis was achieved. While future studies will undoubtedly establish more efficient, or tissue/age-specific promotors, existing promoters are generally effective in cannabis.

### 4.2. Strategies to Improve Gene Transformation Efficiency

Despite advances in the gene transformation of cannabis over the past few years, efficient transgenic regeneration remains an obstacle. The ability to express and introduce transgene and to regenerate de novo shoots or embryos are two important obstacles to producing transgenic cannabis. Recent studies have confirmed that cannabis cells can be efficiently transformed [10,47,176]; however, there is only one report of transgenic cannabis regeneration [93]. The application of genes related to regulating plant growth and development such as WUSCHEL (WUS) [180], BABY BOOM (BBM) [181], and *Growth-Regulating Factors* (GRFs) alone or in combination with *GRF-Interacting Factor* (GIF) [182] is reported as a promising approach to increase plant regeneration efficiency [170]. In this way, ectopic overexpression of genes involved in meristem maintenance, somatic embryogenesis, or phytohormone metabolism can be used to overcome plant regeneration obstacles in recalcitrant plants [183]. Numerous genes involved in meristem maintenance, somatic embryogenesis, and phytohormone metabolism have been identified in different plants [180,183,184]. Accordingly, these studies led to novel approaches for in vitro plant regeneration and *Agrobacterium*-mediated gene transformation research on recalcitrant species. The downside of this approach is that morphogenic genes have adverse pleiotropic impacts and should be removed from transformed or edited plants [180,182,183,184]. The next sections presented recent progress in using morphogenic genes and highlighted possible approaches to overcome negative pleiotropic effects.

#### 4.2.1. Morphogenic Genes, Key Factors in Plant Regeneration

**Figure 7:**
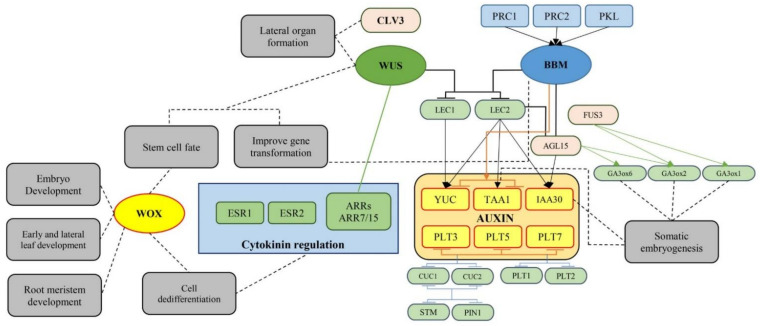
A schematic view of the morphogenic genes demonstrating their roles in plant growth and development as well as in vitro plant regeneration (AGL15: AGAMOUS-LIKE15; ARR: ARABIDOPSIS RESPONSE REGULATOR; BBM: BABY BOOM; CLV3: CLAVATA3; CUC: CUP-SHAPED COTYLEDON; ESR: ENHANCER OF SHOOT REGENERATION; FUS3: FUSCA3; GA3ox: Gibberellin 3-beta-dioxygenase; IAA30: Indole acetic acid inducible 30; LEC: LEAFY COTYLEDON; PIN1: PIN-FORMED 1; PKL: PICKLE; PLT: PLETHORA; PRC: Polycomb repressive complex; STM: SHOOT MERISTEMLESS; TAA: TRYPTOPHAN AMINOTRANSFERASE ARABIDOPSIS; WOX: WUSCHEL-related homeobox; WUS: WUSCHEL; YUC: YUCCA).

Morphogenic genes that induce regeneration, when overexpressed, are categorized into two classes according to their growth reactions. The first group is composed of genes (e.g., SERK1, AGL15, WUS, and STM) that improve a pre-existing response of embryogenesis under in vitro conditions [180]. The second group includes genes (e.g., BBM, EMK, RKD4, LEC1, LEC2, L1L, HAP3A, FUS3, WUS, WOX5, KN1, CUC1, CUC2, ESR1, and ESR2) that stimulate ectopic embryogenesis or meristem induction under in vitro conditions where such events are usually not seen [180,183,185,186]. The roles of morphogenic genes in plant regeneration have been summarized in Figure 7.

In the first class, the overexpression of genes leads to an increase in embryogenesis under in vitro conditions in which the formation of somatic embryos is already observed. For instance, overexpression of *SOMATIC EMBRYOGENESIS RECEPTOR KINASE1* (SERK1) using CaMV 35S promoter resulted in ~4-fold and 2-fold increases in somatic embryogenesis of *Arabidopsis thaliana* [187] and *Coffea canephora* [188], respectively. Similarly, further studies in Arabidopsis [189], cotton [190], and soybean [191] have shown an increase in somatic embryogenesis with overexpression of the *AGAMOUS-LIKE15* (AGL15) gene, a member of the SERK1 protein complex [192]. It is also well documented that SERK1 and SERK3 are the co-receptors of the *Brassinosteroid Insensitive 1* (BRI1) protein. *Brassinosteroid* is a known PGR that plays a key role in plant embryogenesis. The members of SERK proteins seem to act as mediators across the plasma membrane for brassinosteroid signaling [193].

The use of meristem formation-related genes such as *SHOOT MERISTEMLESS* (STM) [194] or *A. thaliana* WUSCHEL (*At*WUS), a key gene for regulating meristem cell fate [195], have also resulted in improving embryogenic responses. Arroyo-Herrera et al. [196] reported that somatic embryogenesis in *Coffea canephora* was increased up to 3–5 fold using an estradiol-inducible *At*WUS construct. In line with this result, Bouchabké-Coussa et al. [197] reported that using the 35S::*At*WUS cassette led to a 3-fold increase in somatic embryogenesis of *Gossypium hirsutum* after *Agrobacterium*-mediated gene transformation. In another study, microspore-derived embryogenesis of *Brassica napus* was increased using the 35S::*Bn*STM construct [198].

In the second class, gene overexpression leads to spontaneous regeneration or direct ectopic regeneration of meristems or embryo-like structures in the lack of inductive in vitro conditions. The early studies have shown that overexpression of the *At*WUS gene [199] and *B. napus* BABY BOOM (*Bn*BBM) gene, a member of the AP2/ERF transcription factors (TFs) [200], leads to embryonic morphogenesis. Consequently, Boutilier et al. [200] reported that the constitutive expression of the *Bn*BBM gene in *A. thaliana* led to ectopic somatic embryogenesis and these ectopic embryos could generate plantlets in the lack of PGRs. This approach has been successfully used to regenerate the transgenic T_0_ plantlets in different plants using orthologs of the *Bn*BBM gene such as transforming 35S::*At*BBM into *Nicotiana tabacum* [201], soybean 35S::*Gm*BBM into *A. thaliana* [202], oil palm 35S::*Eg*BBM into *A. thaliana* [203], and 35S::*Tc*BBM into *Theobroma cacao* [204]. In another study, Tsuwamoto et al. [205] reported that ectopic overexpression of *A. thaliana EMBRYOMAKER* (*At*EMK) led to embryo-like structures at 23% of cotyledon tips; however, these structures could not regenerate the plantlets. They concluded that *At*EMK, a member of the AP2/ERF TFs and related to BBM, should be expressed under a regulated network for avoiding pleiotropic effects and achieving normal plantlets. RKD4 as a key gene in the RWP-RK TF family is another gene that has an important role during early embryo development in *A. thaliana* and plays a pivotal role in the first asymmetrical zygotic division [206,207]. For instance, ectopic somatic embryogenesis of *Phalaenopsis*, a plant typically reluctant to direct somatic embryogenesis, was obtained using the chemical induction of transgenic RKD4 [208].

The overexpression of genes related to embryo maturation (e.g., *FUSCA3* (FUS3), *LEAFY COTYLEDON1* (LEC1), and LEC2) causes similar morphogenic reactions. Lotan et al. [209] characterized the first of these genes namely LEC1, in which a cassette of 35S::*At*LEC1 was transformed to *A. thaliana*. Although the embryo-like structures were produced, functional ectopic embryogenesis was not observed [209]. In another study, Zhu et al. [210] used a 35S::CsL1L cassette for overexpression of *Citrus sinensis* LEC1 after gene transformation in epicotyls of sweet orange and reported that embryo-like structures were obtained after two months. They concluded that overexpression of L1L in Citrus is sufficient for recovering functional somatic embryos. However, Uddenberg et al. [211] indicated that although the overexpression of the LEC1/L1L (PaHAP3A) did not lead to ectopic embryogenesis in vegetative tissues, ectopic embryogenesis was obtained during zygotic embryo maturation. These findings showed that certain types of cells and tissues may be more receptive to morphogenic genes for enhancing ectopic embryogenesis or meristem maintenance [211]. Later studies have shown that the overexpression of LEC2 leads to more somatic embryogenesis in comparison with LEC1, FUS3, or L1L [212,213,214].

However, several studies [190,197,198,200] showed that pre-existing embryogenic responses are increased through overexpression of morphogenic genes, and additional studies revealed that ectopic overexpression of morphogenic genes resulted in improving somatic embryogenesis where such events are usually not seen [183]. Zuo et al. [199] identified the first “meristem” gene in Arabidopsis (*At*WUS) which enhances somatic embryogenesis. They suggested that the vegetative-to-embryonic transition can be stimulated by the WUS gene. Moreover, Gallois et al. [215] demonstrated that the overexpression of WUS and STM genes generates the bulk of cells contiguous to the WUS foci showing primary meristems. In another study, Gallois et al. [216] indicated that unique phenotypes obtained from WUS overexpression were related to different factors such as co-expression of other morphogenic TFs, phytohormone regime, and type of WUS activation (such as GAL4-VP16 activation method and HSP::CRE-mediated excision). Rashid et al. [217] also reported that a member of the WUS/WOX gene family namely *At*WOX5 led to the direct organogenic response in *Nicotiana tabacum*. Luo et al. [218] showed that shoot organogenesis in *N. tabacum* was increased 3-fold by overexpression of the maize STM ortholog *KNOTTED1* (KN1) through 35S::*Zm*KN1 cassette. Similar results were reported by Nishimura et al. [219].

The *CUP-SHAPED COTYLEDON1* (CUC1) and CUC2 genes are known to have a key role in the shoot meristem formation. Daimon et al. [220] indicated that the overexpression of *At*CUC1 and *At*CUC2 under a CaMV 35S promoter resulted in a significant increase (about 6.5–9-fold) in shoot organogenesis of *A. thaliana* transgenic calli. However, no micro-shoots were recovered in the lack of hormones, showing the hormone-dependent function of CUC1 and CUC2 genes. Genes related to the phytohormone signal transduction (both downstream targets and receptors), play a pivotal role in improving plant regeneration; genes like *ENHANCER OF SHOOT REGENERATION 1* (ESR1) and ESR2 that are involved in the cytokinin response pathway and *MONOPTEROS* (MP) that is involved in the auxin response pathway. Several studies have shown that the overexpression of these genes led to an increase in the shoot meristem formation [221,222,223]. Moreover, the phenotypic response of morphogenic genes can be impacted by levels of phytohormones. For instance, Wójcikowska et al. [224] reported that the high and low concentrations of auxin led to callogenesis and somatic embryogenesis, respectively, in the transformed *A. thaliana* overexpressing LEC2 under a dexamethasone (DEX)-inducible system.

#### 4.2.2. Strategies to Overcome Pleiotropic Effects

Based on the aforementioned literature and other references therein, it is clear that the use of morphogenic genes may help to increase regeneration efficiency in plants. However, strong and constitutive overexpression of these genes can lead to unwanted pleiotropic effects such as infertility of regenerated plants [170]. A promising approach to mitigate this is to implement an inducible expression system for these genes in a stable transformation system. This means, it is necessary to apply an additional step that includes optimization of the morphogenic gene expression level with restricting expression after transformation. Such an approach causes enhanced gene transformation and improved fertile and healthy T_0_ plantlets regeneration. Generally, there are five strategies to cope with these challenges including (i) removing the morphogenic gene when no longer needed, (ii) using the inducible expression of the gene for improving morphogenic growth response followed by excision of the inducing ligand for silencing the expression, (iii) using *Agrobacterium*-mediated gene transformation in such a way that promotes the transient expression of the morphogenic genes, (iv) using promoter that can turn off the morphogenic regulatory gene when no longer required, and (v) using GRF-GIF Chimeras [180,182,183,185,186]. 

The excision-based method has provided a reliable strategy for applying morphogenic genes to regenerate fertile and healthy transgenic plantlets. The first successful application of this method through BBM and *Flippase* (FLP)-*recombinase* was reported in *Populus tomentosa* [225]. A T-DNA construct including a single pair of *FLP Recombination Target Sites* (FRT) encompassing both a CaMV 35S promoter stimulating *B. campestris* BBM (*Bc*BBM) expression and a heat-shock inducible promoter stimulating expression of FLP recombinase was designed. Deng et al. [225] reported that after transforming this T-DNA construct, more than 28% of calli produced normal plantlets on phytohormone-free media and also showed that 42 °C heat shock treatment for 2 h resulted in the removal of both the BBM and FLP recombinase cassettes. In another study, Lowe et al. [226] reported that high transformation efficiency in inbred maize was obtained by low expression of *Zm*WUS2 (using the *Agrobacterium NOPALINE SYNTHASE*, or NOS, promoter) and overexpression of *Zm*BBM (using the *Zea mays UBIQUITIN* promoter). The explants were inoculated on dry filter paper to induce a desiccation-stimulated maize promoter derived from an ABA-responsive gene (RAB17) carrying CRE recombinase, which then removed these 3 expression cassettes. After the removal of the BBM, WUS2, and CRE transgenes, only the T-DNA construct with the genes of interest were remained. This method has been successfully used in other recalcitrant plants such as sorghum, rice, corn, and sugarcane [227].

Inducible expression to control morphogenic gene expression is another robust alternative approach. Heidmann et al. [228] reported that gene transformation in *Capsicum annum*, a recalcitrant plant species, was achieved using the 35S::*Bn*BBM~GR vector for gene transformation and cultivation of explants in the medium consisting of DEX and TDZ. In a similar study, Lutz et al. [229] reported that transgenic fertile and healthy *A. thaliana* plantlets were achieved using the DEX-inducible *At*BBM~GR cassette.

The overexpression of *PLANT GROWTH ACTIVATION* genes, such as PGA37 in *A. thaliana* led to the transition of the vegetative phase to the embryogenic phase by using the estradiol-inducible system [230]. The PGA37 gene, based on the DNA-binding domain similarities, encodes the MYB118. Wang et al. [230] reported that root segments developed somatic embryos by PGA37 expression under inducible control, which were related to overexpression of LEC1. The green-yellowish embryonic calli were produced through the expression of PGA37 using the estradiol-inducible system in the medium containing auxin after 7–10 days of inoculation and somatic embryos were obtained after 3–5 weeks. When estradiol was removed from the medium, which causes downregulating PGA37 expression, healthy, fertile plantlets were obtained from the somatic embryos. Moreover, Wang et al. [230] showed that estradiol-induced expression of MYB115, a closely related homolog, resulted in somatic embryogenesis from root segments. A similar strategy using DEX-induced expression of *Tc*LEC2 was successfully used for the regeneration of transgenic somatic embryos in *Theobroma cacao* [231].

Using *Agrobacterium*-mediated gene transformation in such a way that promotes the transient expression of the morphogenic genes is another alternative strategy [232,233,234,235]. Negative selectable markers located outside the T-DNA (beyond the left border) have been used to remove plant cells containing these sequences [233]. Other studies have located a positive marker gene outside the T-DNA, which could be transiently expressed for generating marker-free transgenic plants [236]. A mixture of T-DNAs could be received by placing an *Agrobacterium*-derived isopentyl transferase (IPT) gene outside the T-DNA, with the greater part of the T-strands containing the trait and a minority of T-strands not terminated properly outside the T-DNA, including the flanking IPT gene. Therefore, the transient expression of the IPT gene could stimulate the signaling of cytokinin and subsequently improve shoot proliferation, which results in the recovery of transgenic plants without a selectable marker. This strategy was successfully used in maize genotypes by positioning WUS2 and BBM beyond the left border for transient somatic embryogenesis and subsequent transgenic plantlet regeneration [183]. 

Recently, a new strategy has been developed using the maize *phospholipid transfer protein* (PLTP) promoter driving BBM and the maize *auxin inducible* (AXIG1) promoter for improving gene transformation [237]. The application of these two promoters in the expression cassettes led to somatic embryogenesis within a week, and germination of these somatic embryos within 3–4 weeks. Similarly, the use of a combination of GRF and GIF has been proposed as a new method to tackle negative pleiotropic effects [170,182,238]. Debernardi et al. [170] demonstrated that fertile transgenic wheat, rice, and citrus without obvious developmental defects can be achieved by the expression of a fusion protein combining wheat GRF4 and GIF1. Moreover, they reported that GRF4–GIF1 induced efficient plant regeneration in the absence of exogenous PGRs which helps transgenic plant selection without selectable markers. They also combined CRISPR–Cas9 with GRF4– GIF1 and regenerated edited transgenic plants. Therefore, the combination of CRISPR–Cas9 and GRF4– GIF1 can be considered a powerful and promising strategy for the regeneration of healthy and fertile transgenic plants [170]. This strategy has been recently used in cannabis. Zhang et al. [93] reported that using GRF3– GIF1 in the CRISPR vector resulted in a 1.7-fold increase in edited plant regeneration.

As a future perspective, morphogenic genes can be considered as targets of gene transformation in order to overcome current obstacles in cannabis tissue culture and successful regeneration of transformed plants. Furthermore, useful techniques enabled us to control the side effects of ectopic expression of these genes through transient and inducible gene expression in the host.

### 4.3. Strategies to Prevent Transgene Escape

The frequency of the alleles/genes in a population can be changed due to outcrossing of gametes or gene flow [239]. Thus, transgenes can move from a transgenic plant to their non-transgenic counterparts or wild relatives, a process called transgene escape. It is not uncommon for transgenic plants to mate with their wild relatives. Spontaneous hybridization will occur among transgenic and non-transgenic plants unless the proper distances are maintained [239]. This is an area much of concern, specifically in outcrossing plants, in plant biotechnology. Outcrossing poses negative impacts in terms of contamination in non-transgenic crops but this problem depends on whether a new allele causes an increase in transgene escape or not [240]. While dealing with trans-gene flow one should consider the situations according to transgenic crops [240]. In general, the possible containment and mitigation strategies are physical containment [241], biological/molecular containments (e.g., sterility [242], clistogamy [243], apomixes [244], maternal transformation [245], incompatible genome [246], gene splitting [247], expression in virus [248], genetic use restriction technology (GURT) [239]), and transgenic mitigation [249]. The appropriate approaches should be considered after transgenic cannabis production to prevent transgene escape.

### 4.4. CRISPR/Cas-Mediated Genome Editing

CRISPR/Cas-mediated genome editing has exceptionally improved plant biotechnology [43,250]. This system is robust and offers relatively high target programmability and specificity that can allow accurate genetic modification. The CRISPR/Cas system provides a unique opportunity to improve cannabis varieties with desired traits in a sustainable fashion. Recently, Zhang et al. [93] employed the CRISPR/Cas9 system to knock out the phytoene desaturase gene and they reported that four edited cannabis plantlets with albino phenotype have been successfully generated. However, the numerous new biotechnological methods such as base editing and prime editing based on CRISPR/Cas platforms would expand cannabis synthetic biology and the toolbox of fundamental research. A successful CRISPR/Cas system experiment requires designing target-specific guide RNAs (gRNAs) and an efficient regeneration protocol for developing transgenic/edited plantlets [251]. The gRNA is categorized as a chimeric RNA including a CRISPR RNA (crRNA) and a trans-activating crRNA (tracrRNA). The crRNA consists of a guide (spacer) sequence that accurately navigates the Cas9 protein to the targeted gene. Then, the targeted DNA is cleaved by the RNA-guided DNA endonuclease Cas9. Another key part of CRISPR/Cas-mediated genome editing is the protospacer adjacent motif (PAM), which is a conserved and CRISPR-dependent DNA sequence motif adjacent to the target site (protospacer) and is utilized by the endogenous CRISPR in archaea and bacteria to discriminate invading- and self-DNAs. Generally, the application of an effective and precise CRISPR/Cas system is strongly dependent on the selection of the best guide sequence (gRNA target site) [43,250,251]. 

In the previous sections, promising approaches for developing tissue culture protocols for genetic material delivery (e.g., *Agrobacterium*-mediated method) and regeneration methods have been discussed. In this section, we discuss the principles of designing gRNA in order to produce precise target mutation(s) and prevent off-target mutations as one of the most important prerequisites of genome editing technology. We also present the available bioinformatics tools for designing gRNAs that can be used in cannabis.

**Figure 8:**
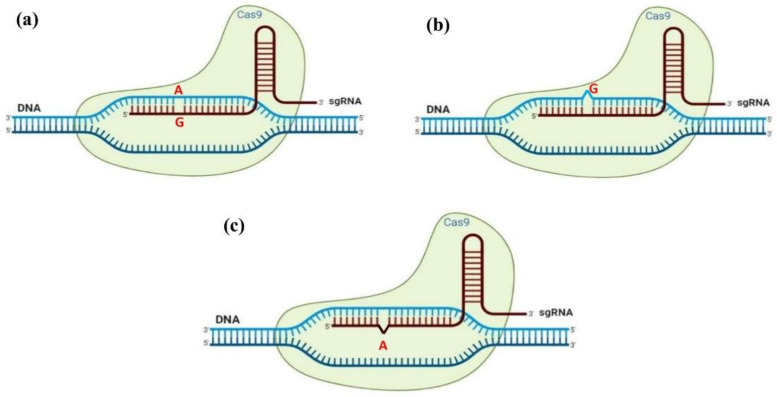
Three types of off-targets induced by CRISPR-mediated genome editing; (**a**) off-target sites with base mismatch, (**b**) off-target sites with extra base (DNA bulge or deletion), and (**c**) off-target site with missing base (RNA bulge or insertion).

The prediction of the presence of off-target sites (i.e., unintended mutations) is one of the most important steps in designing gRNA for CRISPR/Cas-mediated genome editing. The design of the candidate gRNAs starts with the screening of the organism’s whole-genome sequence. Therefore, the availability and accessibility of a high-quality reference genome is required, which is the case in cannabis [252]. The genome of cannabis has been de novo assembled nearly a decade ago [253]; however, there are still some major challenges to using the cannabis reference genome. Currently, 12 different genomes (assembled and annotated) are available for cannabis [252]. Having multiple reference genomes can be considered a bonus, but, contradictorily, there are significant differences in reported genome size, chromosome order, and gene annotations among cannabis genome assemblies [1,2,252]. Therefore, designing a precise and accurate gRNA is an important starting point for high-quality CRISPR/Cas-mediated genome editing in cannabis with the minimum off-target activities. As can be seen in Figure 8, there are three types of off-targets induced by the CRISPR/Cas system including (a) off-target sites with a base mismatch, (b) off-target sites with extra base (DNA bulge or deletion), and (c) off-target site with missing base (RNA bulge or insertion) [254]. Cases (b) and (c) are considered as the indel (insertion or deletion) off-target events [254]. It is necessary to consider these off-targets during genome engineering projects.

Over the past few years, several bioinformatics tools have been developed to design gRNAs and predict, in silico*,* the off-targets. These tools have significantly facilitated the successful application of CRISPR/Cas-mediated genome editing technology [43,250]. A list of bioinformatics tools that contain cannabis genome information is presented in Table 6. 

Generally, these tools exploit a similar algorithm to design gRNA [255]. However, some characteristics related to the gRNA spacer region (e.g., number and type of mismatch, GC-content, length, and indels) and the selection of specific Cas-nucleases vary between these tools [256]. In general, these tools provide multiple sequences for designing gRNA, which are appropriate for editing similar genome conservative motives of various evolutionarily related genotypes [256]. These tools require the sequence of the targeted gene and the Cas-nucleases type. Then they search the genome and provide information related to candidate gRNAs such as the potential protospacers, both non-ranked and ranked with respect to their fitness, and off-target sites [255]. Generally, considering variations in the nucleotide sequences and the increase in individual gene copy number, it is necessary to design multiple gRNAs for each target for an efficient genome editing.

CRISPR RGEN is a web-based tool that also includes BE-Designer [257], Cas-OFFinder [258], Cas-Designer [259], Microhomology-Predictor [260], and some other tools that can be employed for post-editing analysis. Implementation of multiple tools increases the flexibility of CRISPR RGEN Tools that can be illustrated by the following examples: The Cas-Designer can detect potential protospacers in the analyzed sequence. It also provides a broad range of nucleases containing mutant forms with various PAM-sequences: SpCas9 (NRG for the off-target sites and NGG for the target sites), SaCas9 (NNGRRT), StCas9 (NNAGAAW), VRER SpCas9 (NGCG), wtSpCas9 (NNGTGA), NmCas9 (NNNNGMTT), VQR SpCas9 (NGA), CjCas9 (NNNNRYAC), CjCas9 (NNNVRYAC), BhCas12b (TTN), FnCpf1 (KYTV), FnCpf1 (TTN), AsCpf1 (TTTV), AsCpf1 (TTTN), etc. In the protospacer searches, one may specify if it is essential to find the probability of single nucleotide indels. In this case, the tool notifies about enhancing the time of the search. However, if this option is not applied, the results of the search returns immediately. Other parameters are chosen by default. Cannabis genome should be selected from the list to run the search. Further changes to other options of the Cas-Designer can be applied when it is locally installed on a computer [255]. The Microhomology-Predictor facilitates the forecast of the type of microhomology-mediated end-joining (MMEJ) and nonhomologous end-joining (NHEJ) by analyzing the microhomology of nucleotide sequences in the sites of double-strand breaks [260]. The Cas-OFFinder tool can be used to screen possible off-target sites in the genome. Furthermore, this tool can be independently applied for previously selected gRNAs to find off-target sites. For analysis, it is essential to insert the selected gRNAs sequences, specify the number of indels (both in RNA and DNA) and the adequate number of mismatched nucleotides, similarly Cas-Designer [258]. The BE-Designer tool can be applied for designing gRNAs to edit individual nitrogenous bases. Although Cas-Designer works similarly to BE-Designer, there are some differences. For instance, the selection of the corresponding nucleases and the PAM-regions is slightly limited in the BE-Designer, and the “window” size should be chosen for searching the targeted substitution of nucleotides (A→G or C→T) and the editing sites. The results would be provided in a table, in which the substituted nucleotides are shown with a specific color [255].

The CRISPOR [261] is another user-friendly tool with a detailed user manual. Selecting optimal protospacers in this tool can be performed in three steps. In the first step, the sequence of the targeted gene should be provided by the user. In the second step, the genome (e.g., cannabis) should be selected. In the third step, the Cas9 nuclease must be selected out of 33 options. The results of the search are provided in both table and graphic formats. The analyzed sequence is represented, and the potential edited sites are highlighted with various colors, relating to low, medium, and high specificity. Moreover, the table of the results includes information regarding the “Cloning/PCR primers”, protospacers sequences, their positions with indicated DNA chains, expression of a specific gRNA, and cloning. Two algorithms are used to assess the efficiency of the proposed gRNAs and, also, specificity is used to rank the proposed gRNAs. In addition to predicting the types of microhomology, it foresees the off-target sites with the number of mismatches [262].

The CRISPR/Cas9 Target online predictor (CCTop) tool [263] is applied to design and screen gRNAs for on-target and off-target sites. It can use a wide range of Cas-9 nucleases from various bacterial strains (e.g., *Campylobacter jejuni*, *Streptococcus pyogenes*, *Treponema denticola*, *Staphylococcus aureus*, *Streptococcus thermophilus*, and *Neisseria meningitidis*), their mutant forms, as well as Cpf1 from *Francisella novicida* and *Lachnospiraceae/Acadaminoccus*. The most intriguing feature of this tool is its ability to add nucleotides in the form of one or two guanines at the 5′-end of the spacer region to improve in vitro transcription. Additionally, two favorable nucleotides adjacent to the PAM region can be considered in order to prevent them from being with the gRNA. This tool exploits the CRISPRater prognosis algorithm to rank predicted gRNAs. gRNAs in intergenic, introns, and exons spacers are presented in various colors. This tool shows information in both table and graphic formats [255].

The CHOPCHOP tool [264] can be used to design and screen gRNAs with the capability to work in complex with the Cpf1 nuclease and Cas9-nuclease and its nickase forms. The search for gRNA can be run either in all 5′- or 3′-non-coding regions, splicing sites, exons, and in the promoter sequence or in a specified coding region, the length of which would be chosen individually. Moreover, the restriction sites in the edited region can be screened by the corresponding enzymes with the manufacturer’s name. After finishing the search, an adjustable color graphic and an interactive table with ranked gRNA target sequences, consisting of DNA chains, the GC-composition, and other information are provided [255].

The Breaking Cas tool [265] can be applied for designing gRNAs and screening the off-target sites. It is essential to select the form of nuclease from the three types of Cpf1 and the four types of Cas9 with mismatch number and the length of pre-set spacers. It is also possible to select a “Custom” nuclease, which is followed by providing the corresponding lengths of the spacer sequence of gRNA, PAM-sequences, and an acceptable number of mismatches. 

**Figure 9:**
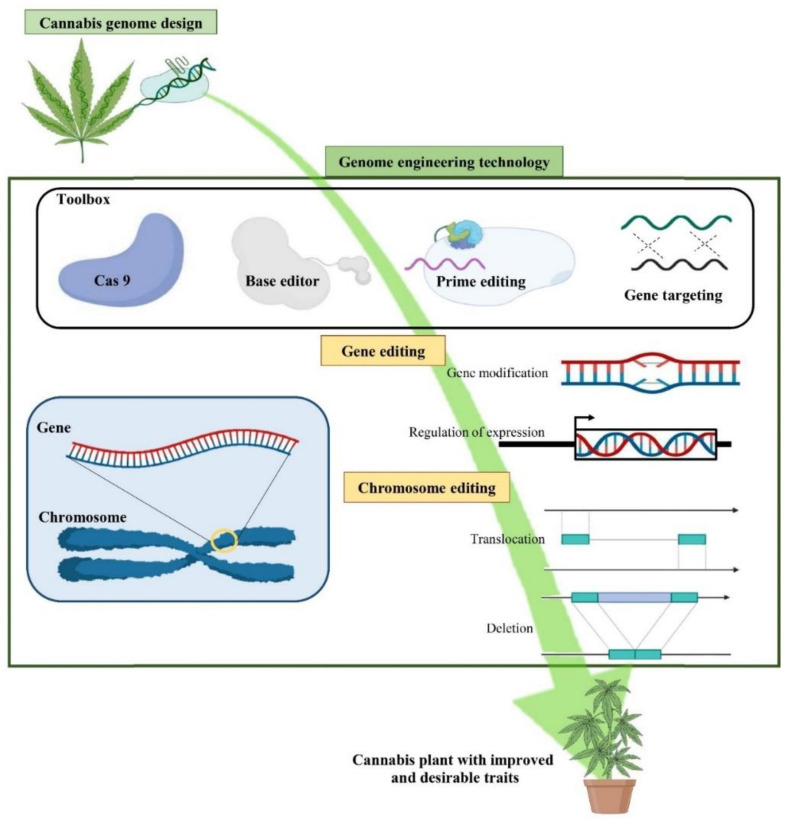
A schematic view of Cannabis genome engineering.

All in all, different types of CRISPR-mediated genome editing systems, including targeted mutagenesis to knock out a target gene [266], base editing systems for transition substitution using a combination of Cas9 nickase and either adenosine or cytidine deaminase [267], transversion substitution using Cas9 nickase, cytidine deaminase and uracil DNA glycosylase [268], as well as prime editing systems using DNA or RNA as donors [269], have been recently developed and applied in different species [270]. Very recently the application of the CRISPR system [93] and prediction systems through big data analysis and machine learning [147] have been reported in cannabis. Focusing on cannabis genome engineering, the design of genome sequences is as essential as the applied genome-editing technology. When the target gene is epigenetically regulated, not only genome sequences but also histone modifications, DNA methylation, and chromatin structure, may need to be modified to generate the desired cannabis plant. Furthermore, systems for highly efficient sequence-specific nuclease delivery and subsequent genome-edited cell and plant selection must also be developed to accelerate the breeding of designer cannabis plants (Figure 9).

## 5. Conclusions and Future Objectives

Efficient and reliable in vitro culture procedures can be considered as an important prerequisite for successful gene transformation, genome editing, micropropagation, and conservation of cannabis. Micropropagation is a powerful tool that can develop and propagate new varieties. Polyploidy induction is also a method that can influence secondary metabolites. Although current shoot proliferation-based protocols are relatively developed for mass propagation of cannabis, it is necessary to develop somatic embryogenesis, organogenesis, and haploid production protocols especially for gene transformation and genome editing studies. *Agrobacterium*-mediated gene transformation has been described in cannabis. Recently, transgenic plant regeneration has been successfully obtained. It seems that using morphogenic genes can help overcome challenges in transgenic plant regeneration. The production of secondary metabolites is another important aspect of in vitro culture of cannabis. Although several studies have attempted to manipulate secondary metabolite production in cell suspension culture and hairy root culture of cannabis, small amounts of cannabinoids have been produced. Therefore, it is necessary to apply new methods such as CRISPR/Cas-equipped *A. rhizogenes*-mediated hairy root culture for increasing or changing secondary metabolite profiles. Machine learning algorithms can also be considered as robust computational biology for a comprehensive study of secondary metabolites and, also, modeling and optimizing hairy root culture or cell suspension culture for improving its yield. Generally, cannabis plants still provide the most efficient natural sources of secondary metabolites, and, in particular, cannabinoids and modern biotechnologies will play an important role in further genetic improvement. 

## Figures and Tables

**Table 1:** Some pharmacological properties of Cannabis.

Secondary Metabolite	Structure	Medicinal Effects	References
Tetrahydrocannabinol	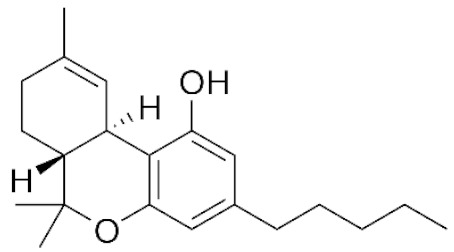	Anti-inflammatory, antispastic, analgesic, antineoplastic, antiemetic activity, antipruritic agent, bronchodilator	Maayah et al. [12] and Workman et al. [13]
Cannabidiol	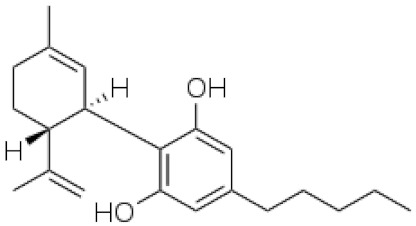	Anticonvulsant, antipsychotic, analgésic and anti-inflammatory, neuroprotection, antibacterial, antiemetic, anxiolytic, immunomodulator, antidepressant, cytotoxic for some cancer cell lines	Maayah et al. [12], Cassano et al. [14], and Alves et al. [15]
Cannabinol	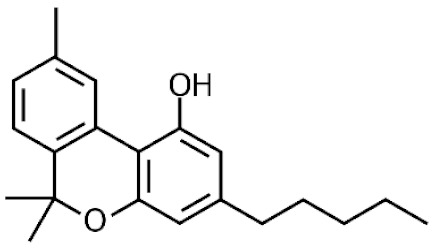	Anti-inflammatory, antibacterial, anticonvulsant	Maayah et al. [12] and Alves et al. [15]
Cannabigerol	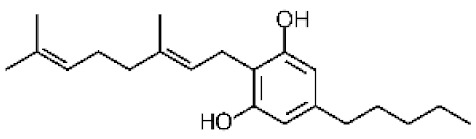	Analgesic, antifungal, antibacterial, antitumor activity, decreasing intraocular pressure	Cassano et al. [14] and Alves et al. [15]
Tetrahydrocannabivarin	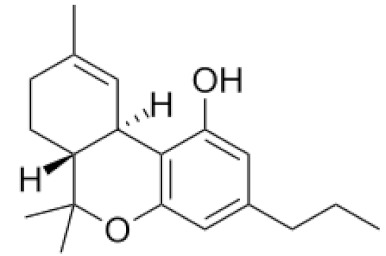	Anti-inflammatory, anti-nociceptive, anticonvulsant	Maayah et al. [12], Workman et al. [13], and Cassano et al. [14]
Myrcene	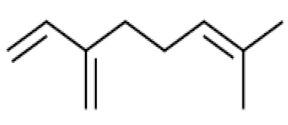	Anti-inflammatory, anti-nociceptive, antioxidative	Baron [16], Nuutinen [17], and Hwang et al. [18]
β-caryophyllene	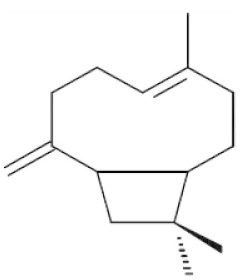	Anti-inflammatory, anti-convulsive, the astrocyte activation, inhibition of microglia, modulate nociception, neuroprotection, dopaminergic cell protection, addiction and alcohol consumption, feeding behavior, preventing alcohol-induced damage	Baron [16], Nuutinen [17], and Aly et al. [19]
Caryophyllene oxide	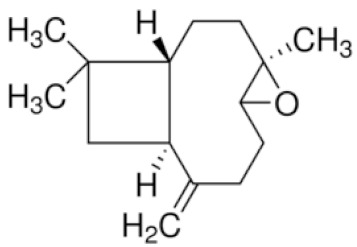	Anticancer, antioxidant, bactericide, and analgesic	Baron [16], Nuutinen [17], and Ciftci et al. [20]
Humulene	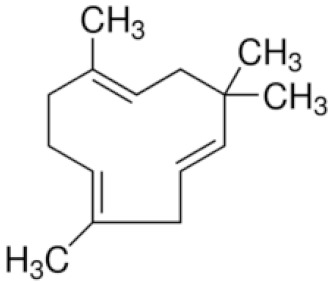	Treatment of depression, insomnia, nervousness, anxiety, delirium, and digestive disorders	Baron [16], Nuutinen [17], and Shah et al. [21]
α-Pinene	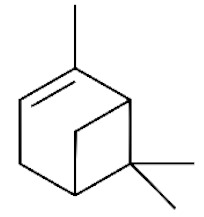	Anti-tumor, anti-allergic bronchodilator, anti-metastatic, antioxidant, anti-inflammatory, anxiolytic, and hypnotic	Baron [16], Nuutinen [17], and Kołodziejczyk et al. [22]
β -Pinene	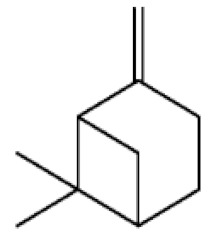	Treatment of cancer, diabetes, atherosclerosis, and obesity	Baron [16], Nuutinen [17], and Guzmán-Gutiérrez et al. [23]
Linalool	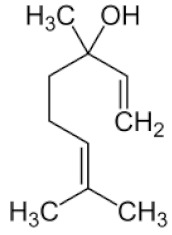	Antioxidative, anti-nociceptive, neuroprotective, anticonvulsant, anti-inflammatory, sedative, anti-microbial, anti-depressant, hepatoprotective, anti-tumor	Baron [16], Nuutinen [17], and Jana et al. [24]
Limonene	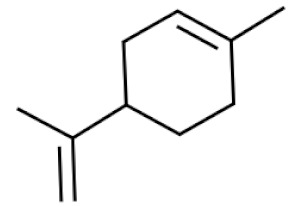	Anti-tumor, anticancer, ameliorate depression, stress, inflammation, viral infections, and spasms	Baron [16], Nuutinen [17], and Srividya et al. [25]
Perillyl alcohol	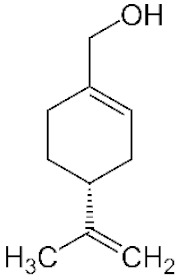	Anti-inflammatory, anticancer, antioxidant, anti-tumor, nociceptive, antifungal, hepatoprotective, anti-parasitic	Baron [16], Nuutinen [17], and Faria et al. [26]
Terpinolene	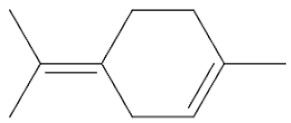	Anti-inflammatory, antioxidant, anti-nociceptive	Baron [16], Nuutinen [17], and Zhao et al. [27]
γ-Terpinene	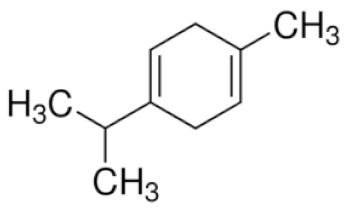	Anti-nociceptive and anti-inflammatory	Baron [16], Nuutinen [17], and Castro et al. [28]
α -Terpinene	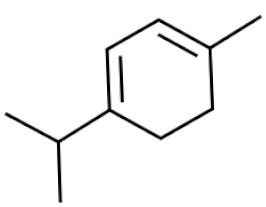	Antioxidant and antibiotic	Baron [16], Nuutinen [17], and de Oliveira et al. [29]
Terpineol	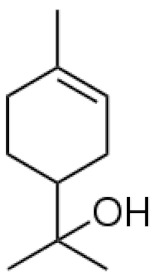	Anticancer, spasmolytic, anti-tumorigenic, antibiotic, anti-inflammatory, anticonvulsant, treatment of spasms, neurological damages, pain, and asthma	Baron [16], Nuutinen [17], and Vieira et al. [30]
Geraniol	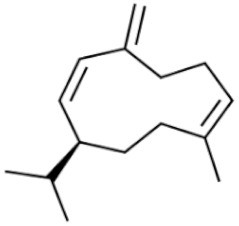	Treatment of depression, cancer, cardiac dysfunction, pain, colitis, neuropathy, atherosclerosis, allergic asthma, inflammation, tissue injuries, PD, and diabetes.	Baron [16], Nuutinen [17], and Lira et al. [31]
Nerolidol	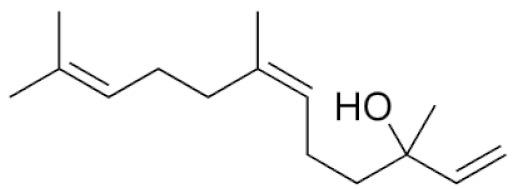	Antioxidant, anti-inflammatory, anticancer, sedative, fungicide, anxiolytic, bactericide, anti-parasitic, antidepressant, and antinociceptive.	Baron [16], Nuutinen [17], and Barros Silva Soares de Souza et al. [32]
Borneol	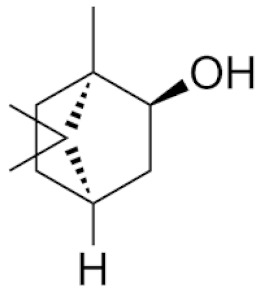	DNA preserving, antipyretic, anti-inflammatory, neuroprotective, antioxidant, and anti-nociceptive.	Baron [16], Nuutinen [17], and Yang et al. [33]
α -Bisabolol	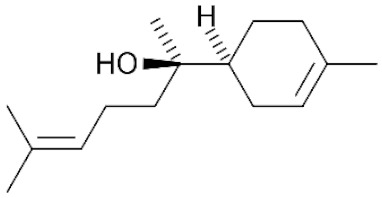	Anti-parasitic, anti-inflammatory, anti-nociceptive, anticancer, antibiotic, anti-tumor, and anti-apoptotic.	Baron [16], Nuutinen [17], and Xu et al. [34]
Bisabolenes	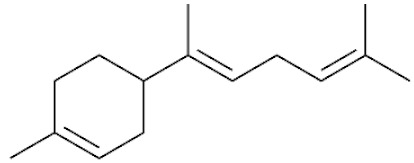	Anti-bacterial, anticancer, anti-convulsive, and anti-tumor.	Baron [16], Nuutinen [17], and Gogineni et al. [35]
β -elemene	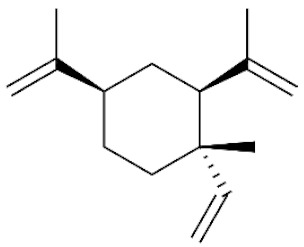	Anti-inflammatory, treatment of cancer, liver fibrosis, atherosclerosis, and MS.	Baron [16], Nuutinen [17], and Tong et al. [36]
Fenchone	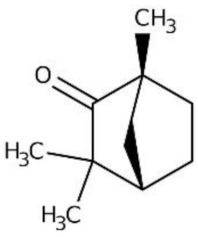	Antimicrobial, anticancer, and anti-tumor.	Baron [16], Nuutinen [17], and Müller et al. [37]
Pulegone	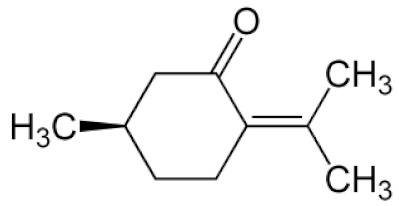	Antimicrobial, anticancer, and anti-tumor.	Baron [16], Nuutinen [17], and Yang et al. [33]
α -Phellandrene	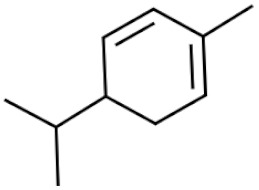	Pro-apoptotic, antimicrobial, anti-inflammatory, anti-depressive, immunomodulatory, and anti-nociceptive.	Baron [16], Nuutinen [17], and de Christo Scherer et al. [38]
β -eudesmol	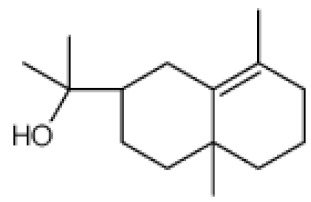	Anti-convulsant, anti-inflammatory, and anticancer.	Baron [16], Nuutinen [17], and Kotawong et al. [39]

**Table 2:** In vitro regeneration studies in cannabis.

Genotype(s)	Explant(s)	Morphogenetic Response(s)	Media, PGRs (mg/L), and Additives	Culture Conditions (Temperature, Light Intensity, etc.)	Outcomes and Descriptions	Reference
OSU	Roots derived from in vitro grown seedling	Cell suspension cultures	Gamborg’s medium (67-V), 2,4-D (1.5) + NAA (0.1) + IAA (1) + Kin (0.25) + casein hydrolysate (1)	Light at 26 °C, light condition: NR	The maximum callogenesis was observed in the media containing 0.1 mg/L NAA + 0.25 mg/L Kin + 1 mg/L casein hydrolysate.	Veliky and Genest [62]
C-71, TU-A	Leaves, hypocotyl, root, and female and male floral parts	Callogenesis	MS +2,4-D (1) + Kin (0.01–0.1)	Light at 26 °C, light condition: NR	The maximum callogenesis was observed in MS medium supplemented with 1 mg/L 2,4-D + 0.1 mg/L Kin.	Itokawa et al. [63]
C-150, C-152	Bracts, calyx	Callogenesis	Miller’s medium + Murashige’s iron source + IAA (0.25, 1) + NAA (0.1, 0.25) + 2,4-D (0.2) + Kin (1, 1.5, 2) + casein hydrolysate (1)	12 h photoperiod (~700 lx); temperature: NR	The maximum callogenesis in different cultivars and explants was observed in the media containing 0.5 mg/L NAA + 2 mg/L Kin. Although root formation was observed on the surface of the callus, it was inhibited by using 0.2 mg/L 2,4-D.	Hemphill et al. [49]
OSU	Leaf, roots, and stem	Callogenesis and cell suspension cultures	MS salts +B5 vitamins medium + 2,4-D (0–5), 2,4,5-T (0–5), NAA (0–5), kin (0–5), 2iP (0–5), and BAP (0–5)	Light at 26 °C, light condition: NR	Callogenesis in stem segments was observed in 0.5 mg/L 2,4-D and 0.1 mg/L BAP. 2,4,5-T and NAA could not produce calli in stem segments. Callogenesis in root segments was observed in 0.1–1 mg/L NAA and 5 mg/L kin. as well as 5 mg/L BAP and 1 mg/L NAA. 2,4,5-T and 2,4-D could not produce calli in root segments. Generally, the response of Cannabis explants to PGRS was significantly affected by the type of explant. The maximum cell masses in cell suspension culture were produced in 3 mg/L 2,4,5-T without subculture.	Loh et al. [50]
OSU	Different parts of seedling	Callogenesis and cell suspension cultures	MS salts +B5 vitamins medium +2,4-D (0.1) + Kin (0.5)	Light at 27 °C, light condition: NR	Six to eight weeks after culturing, callogenesis was obtained. The maximum cell masses in cell suspension culture were produced in 3 mg/L 2,4,5-T.	Hartsel et al. [64]
F56 and F77	Apical and axillary buds	Shoot organogenesis and in vitro rooting	MS + IBA (0–20) + BAP (0.45) + 3% glucose + 1% sucrose + charcoal (0–2 g/L)	27 ± 2 °C under 16 h photoperiod (360 µmol/m^2^/s)	The highest shoot regeneration was observed in 2 mg/L IBA + 0.45 mg/L BAP + 3% glucose + 1% sucrose. The maximum root regeneration was observed in 20 mg/L IBA + 2g/l charcoal.	Richez-Dumanois et al. [65]
NR	Leaf	Cell suspension cultures	B5 medium + 1 mg/L 2,4 -D + 0.5 mg/L KIN + 3% glucose	Darkness at 25 °C	A cell suspension culture of Cannabis was able to convert CBD to bound CBE and THC to CBC.	Braemer and Paris [51]
Sud Italian	Leaf, hypocotyl, cotyledon, and root	Callogenesis and shoot regeneration	MS salts +B5 vitamins medium +2,4-D (3–10) + BAP (0.01–1)	27 ± 2 °C under 16 h photoperiod (360 µmol/m^2^/s)	Although all explants produced callus, the maximum callogenesis was observed in leaf and hypocotyl segments. The maximum shoot regeneration was obtained from hypocotyl segments; however, leaf explants could not produce shoots.	Mandolino and Ranalli [66]
Silesia, Juso-15, Novosadska, Fibrimon-24, and Fedrina-74	Leaves, petioles, internodes, and axillary buds	Callogenesis, shoot regeneration, and in vitro rooting	MS + 2,4-D (2 and 4), DIC (2 and 3), NAA (0.5, 1 and 2), and Kin (1, 2, and 4)	22 °C under a 16 h photoperiod (~2000 lx)	Callogenesis and shoot regeneration responses were varied based on different explants and genotypes. The highest callogenesis was obtained by petiole segments of cv. Fibrimon-24. The maximum indirect shoot regeneration was observed on a medium containing DIC. In vitro rooting was obtained from 1.0 mg/L IAA and 1.0 mg/L NAA.	Slusarkiewicz-Jarzina et al. [67]
Beniko and Bialobrzeskie	Stems, roots, and adventitious shoots	Direct organogenesis and indirect embryogenesis	Knapp’s medium + BAP (NR) + NAA (NR) + IAA (NR)	NR	After two weeks direct organogenesis was observed. Somatic embryos were also obtained from the medium containing NAA and BAP along with 500 mg/L activated charcoal.	Plawuszewski et al. [68]
Finola	Lateral buds	Shoot regeneration and in vitro rooting	Shoot regeneration: MS + TDZ (0.1–0.5) + NAA (0.05–0.3) Rooting: 1/2MS or MS + IBA (0.01–0.5) + NAA (0.01–0.25)	25 °C under a 16 h photoperiod (~3000 lx)	The maximum shoot regeneration was observed in 0.35 mg/L TDZ + 0.3 mg/L NAA. The highest root formation was observed in MS + 0.2 mg/L IBA + 0.15 mg/L NAA.	Bing et al. [69]
Bialobrzeskie, Silesia, and Beniko	Cotyledons, stems, and roots	Callogenesis, shoot regeneration, and in vitro rooting	Knopp’s medium + Kin (1), BAP (0.2), NAA (0.03–0.05), IAA (2)	24–26 °C under a 16 h photoperiod (light intensity: NR)	Callogenesis and shoot regeneration responses were varied based on different explants and genotypes. The highest callogenesis was obtained from 1 mg/L Kin and 0.05 NAA mg/L. The maximum indirect shoot regeneration was observed in a medium containing 0.2 mg/L BAP and 0.03 mg/L NAA. In vitro rooting was obtained from 2.0 mg/L IAA.	Wielgus et al. [70]
Changtu	Shoot tips	Shoot proliferation and in vitro rooting	Shoot regeneration: MS + BAP (1.0, 2.0, 5.0), Kin (1.0, 2.0, 5.0), TDZ (0.1, 0.2, 0.5), NAA (0.05, 0.1, 0.5) Rooting: 1/2MS, MS, B5 or NN + NAA (0.05, 0.25), IAA (0.05, 0.25), IBA (0.1, 0.5)	25 ± 1 °C under a 16 h photoperiod (2500 lx)	The highest shoot proliferation was obtained from 0.2 mg/L 1TDZ and 0.1 NAA mg/L. The highest in vitro rooting was obtained from MS + 0.1 mg/L IBA + 0.05 mg/L NAA.	Wang et al. [71]
MX-1	Nodal segments containing axillary buds	Shoot proliferation and in vitro rooting	Shoot regeneration: MS + BAP (0.5–9 μM), Kin (0.5–9 μM), TDZ (0.5–9 μM), GA (0.7 μM) In vitro rooting: 1/2MS + 500 mg/L activated charcoal + IAA (2.5, 5 μM), IBA (2.5, 5 μM), NAA (2.5, 5 μM)	25 ± 2 °C under a 16 h photoperiod (52 µmol/m^2^/s)	The highest shoot proliferation was obtained from 0.5 μM TDZ. The highest in vitro rooting was obtained from 2.5 μM IBA.	Lata et al. [72] and Lata et al. [73]
MX_E-1_	Leaf	Callogenesis, shoot organogenesis, and in vitro rooting	Callogenesis: MS + 1.0 μM TDZ + (0.5, 1.0, 1.5, and 2.0 μM) of IAA, NAA, IBA Shoot organogenesis: MS + (0.5, 1.0, 2.5, 5.0, and 10.0 μM) of BAP, Kin, TDZIn vitro rooting: 1/2MS + (0.5, 1.0, 2.5, 5.0, and 10.0 μM) of IAA, IBA, and NAA	25 ± 2 °C under a 16 h photoperiod (52 µmol/m^2^/s)	The maximum callogenesis was obtained from 0.5 μM NAA + 1.0 μM TDZ. The highest shoot organogenesis was observed in 0.5 μM TDZ. The highest in vitro rooting was obtained from 2.5 μM IBA.	Lata et al. [74]
NR	Cotyledon and epicotyl	Indirect shoot organogenesis, and in vitro rooting	Indirect shoot organogenesis: MS +BAP (0.1, 0.2, 0.5, 1, 2, 3), IBA (0.5), TDZ (0.1, 0.2, 0.5, 1, 2, 3), IAA (0.5) In vitro rooting: MS + IBA (0.1, 0.2, 0.5, 1) + NAA (0.1, 0.2, 0.5, 1)	NR	The maximum callogenesis was obtained from cotyledon explants in MS medium supplemented with 3 mg/L TDZ + 0.5 mg/L IBA. The maximum shoot organogenesis was achieved from epicotyl segments in MS medium supplemented with 2 mg/L BAP + 0.5 mg/L IBA.	Movahedi et al. [75]
NR	Leaf and hypocotyl	Indirect shoot organogenesis, and in vitro rooting	Callogenesis and shoot regeneration: MS+ 2,4-D (0.1, 0.2, 0.5, 1), NAA (0.5, 1, 2, 3), BAP (0.5) In vitro rooting: MS+ (0.1, 0.2, 0.5, 1) of IBA and NAA	25 °C under a 16 h photoperiod (light intensity: NR)	The maximum callogenesis was observed from leaf segments in 1 mg/L 2,4-D + 0.5 mg/L BAP. However, indirect organogenesis was only obtained from hypocotyl explants in the medium containing 0.1 mg/L 2,4-D + 0.5 mg/L BAP. Successful in vitro rooting was observed in all of the treatments.	Movahedi et al. [76]
NR	Leaf and hypocotyl	Callogenesis	Callogenesis and shoot regeneration: MS+ BAP (0.1, 0.2, 0.5, 1, 2, 3), TDZ (0.1, 0.2, 0.5, 1, 2, 3), IBA (0.5)	25 °C under a 16 h photoperiod (light intensity: NR)	The maximum callogenesis was obtained from MS medium containing 0.5 mg/L IBA + 2 mg/L TDZ using leaf segments. Indirect shoot formation was observed on various concentrations of BAP in hypocotyl segments.	Movahedi et al. [77]
Mexican variety	Nodal segments containing axillary buds	Shoot proliferation and in vitro rooting	Shoot regeneration: MS + 500 mg/L activated charcoal + TDZ (0.05, 0.50, 1, 2, 3, 4, and 5 μM), mT (0.05, 0.50, 1, 2, 3, 4, and 5 μM) In vitro rooting: 1/2MS + 500 mg/L activated charcoal + IBA (0.05, 0.50, 1, 2, 3, 4, and 5 μM)	25 ± 2 °C under a 16 h photoperiod (52 µmol/m^2^/s)	2 μM mT resulted in the highest shoot regeneration and in vitro rooting.	Lata et al. [78]
Kunming, Neimeng 700, YM535, Anhui727, DaliS1, Heilongjiang698, Heilongjiang449, BM2	Cotyledons	Shoot regeneration and in vitro rooting	Callogenesis: MS +BAP (4,6,8), ZT (0.5, 1, 1.5), TDZ (0.1, 0.2, 0.4), NAA (0.2, 0.4, 0.6) Shoot organogenesis: MS + TDZ (0.1, 0.2, 0.3, 0.4, 0.5), NAA (0.2, 0.4, 0.6) In vitro rooting: 1/2MS + IBA (0.2, 0.5, 1, 2)	22 ± 2 °C under a 16 h photoperiod (36 µmol/m^2^/s)	While BA and ZT produced Soft, flaky, green and yellow callus, TDZ produced Hard, green and nodular callus. The maximum shoot regeneration was obtained from 0.4 mg/L TDZ + 0.2 mg/L NAA. The regenerated micro-shoots had a high vitrification rate and a low chance of survival in the rooting step when higher than 0.5 mg/L TDZ was used. Shoot regeneration responses were varied based on cotyledon age and genotypes. The juvenile cotyledon (2-day-old) showed the best regeneration potential.	Chaohua et al. [79]
1KG2TF, S1525, H5458	Immature and mature inflorescences	Shoot regeneration	Shoot organogenesis: MS + TDZ (0.1, 2, 5, 10)	23 °C under a 16 h photoperiod (10–30 µmol/m^2^/s)	Shoot regeneration was observed in 1 and 10 μmol TDZ. MS+ 0.03% also activated charcoal+ 1.86 μmol kin+ 0.54 μmol NAA resulted in shoot multiplication and in vitro rooting.	Piunno et al. [80]
Bialobriezskie, Tygra, Fibrol, Monoica, USO-31	Cotyledonary node, epicotyl with first node, hypocotyl, epicotyl with first and second node, shoot apical meristem, and shoot apex	Shoot regeneration	MS + 9.31 μg/L NAA + 0.23 mg/L BAP + mT (1–5), BAP9THP (1–5), PEO-IAA (10 μmol/l)	19 °C under a 16 h photoperiod (56 µmol/m^2^/s)	Epicotyl with the first node resulted in the highest shoot regeneration. The maximum shoot regeneration was also observed in the medium containing BAP9THP.	Smýkalová et al. [81]
U91, GRC, U37, RTG, U82, U42, U22, U38, U31, and U61	Leaf	Callogenesis	MS and DKW + NAA (0.5 μM), TDZ (0.5 and 1 μM)	25 °C under a 16 h photoperiod (10–41 ± 4 µmol/m^2^/s)	Although 1.0 μM TDZ + 0.5 μM NAA produced callus in all genotypes, callogenesis was determined to be species-specific.	Monthony et al. [82]
E1, E4, and E40 of Epsilon 68	Nodal segments containing axillary buds, shoot tips	Shoot regeneration and in vitro rooting	Shoot regeneration: MS+ BAP (0.5–2), TDZ (0.1–0.5), mT (0.1–1) In vitro rooting: 1/2 MS + (0.25, 0.5, 0.75) of IBA and IAA	25 ± 1 °C under a 18 h photoperiod (60 µmol/m^2^/s)	The highest shoot regeneration was observed in the media containing 1–2 mg/L ZEA^RIB^ + 0.02 mg/L NAA.	Wróbel et al. [83]
Felina32, Ferimon, Fedora17, Finola, and USO31	Leaves, hypocotyl, and cotyledon	Direct shoot regeneration	MS+ BAP (0.5, 1, 2), TDZ (0.4, 1), NAA (0.02, 0.2), IBA (0.5), 2,4-D (0.1), 4-CPPU (1.0), ZT^RIB^ (1, 2), BAP^RIB^ (1)	22 ± 1 °C under a 16 h photoperiod (90.15 µmol/m^2^/s)	Cotyledon and leaf explants had poor shoot regeneration responses, while hypocotyl segments were the best explant for shoot regeneration.	Galán-Ávila et al. [84]
U82 and U91	Inflorescences (single florets vs. pairs of florets)	Direct shoot regeneration	DKW + BAP (0.0, 0.01, 0.1, 1.0, and 10 μM) for both cultivars DKW + mT (0.0, 0.01, 0.1, 1.0, and 10 μM) for U91 cultivar	25 °C under a 16 h photoperiod (50 µmol/m^2^/s)	Floral reversion was observed in the meristematic florets. These explants can be applied to improve regeneration frequency. Although the pairs of florets had a significant effect on the reversion rate and production of healthier plantlets, PGRs and cultivars had no remarkable impact on the reversion rate.	Monthony et al. [85]
Aida, Juani, Magda, Moniek, Octavia, and Pilar	Axillary buds	Shoot regeneration	MS, B_5_ with vitamins (Formula βA), and B_5_ without MS vitamins (Formula βH) + 2μM mT, 2μM IBA NAA, 2μM IBA	25 ± 0.5 °C under a 18 h photoperiod (50 µmol/m^2^/s)	Both Formula β media resulted in a better response. Also, results showed that success was cultivar-dependent.	Codesido et al. [86]
MX-CBD-11 and MX-CBD-707	Axillary buds	Shoot regeneration	MS + TDZ (0.011, 0.1, 0.11, 0.22, 0.44, 0.88, 1.76 mg/L), mT (0.012, 0.12, 0.24, 0.48, 0.5, 0.96, 1.93 mg/L), BAP (1, 2.5, 5 mg/L), IAA (0.1 mg/L)	25 °C under a 16-h photoperiod (light intensity: NR)	The results showed that the type and concentration of PGRs and genotype had a significant effect on cannabis shoot regeneration. MS medium supplemented with 0.1 mg/L TDZ also resulted in the highest regeneration frequency in both genotypes.	Mubi et al. [87]
a high CBD and a high CBG	Axillary buds	Shoot regeneration	Shoot regeneration: Full- or half-strength MS + BAP (1.0, 2.0, 4.0 and 8.0 μM), TDZ (1.0, 2.0, 4.0 and 8.0 μM) In vitro rooting: Full- or half-strength MS + IBA (1.0, 2.0, 4.0 and 8.0 μM), NAA (1.0, 2.0, 4.0 and 8.0 μM)	23 ± 1 °C under a 16 h photoperiod (50 µmol/m^2^/s)	Both full and half-strength MS + 4.0 μM BA resulted in the maximum shoot number and shoot length in both genotypes. The highest root formation was also obtained from both full and half-strength MS + 4.0 μM IBA or NAA.	Ioannidis et al. [88]
Hemp cultivars (Wife and Dinamed CBD)	Stem tips	Shoot proliferation	MS, MS + Mesos components, 2.5× MS with vitamins, MS with vitamins + added Mesos, MS with vitamins + added vitamins, MS with vitamins + added Mesos and vitamins; MS with vitamins + added Mesos and vitamins + NH_4_NO_3_ (0, 500, 1000, or 1500 mg/L)	25 °C under a 18 h photoperiod (40 µmol/m^2^/s)	The maximum shoot multiplication, leaf lamina development, and shoot extension were observed in MS with vitamins + added Mesos and vitamins + 500 mg/L NH_4_NO_3_. 75% to 100% ex vitro rooting was also obtained in Rockwool.	Jessica et al. [89]
US Nursery Cherry 1	Apical shoot tip and single node	Shoot proliferation	DKW without PGRs	23 ± 2 °C under a 14 h photoperiod (25, 46, 85, 167 µmol/m^2^/s) in vessels with vented or non-vented closures	The maximum number of harvested shoot tips was observed in 46 µmol/m^2^/s in non-vented vessels.	Murphy and Adelberg [90]
BCN Power Plant, Safari Cake 747, CD13, and Blue Widow	Stem segments	Shoot growth and development	Safari Flower (SF) vegetative fertilizer solution + Sigma-Aldrich Canada + ethanesulfonic acid + 5-mM MES (2-(Nmorpholino)	22 ± 3 °C under a 18 h photoperiod (50, 100, 150 µmol/m^2^/s)	The roles of Rockwool medium pH, cutting length, the moisture content in the vessels, basal wounding methods, the capacity of culture vessel gas exchange, and light intensity were studied. The percent of rooted plants was increased by using both 5- and 7-cm explant lengths compared to 3-cm explant length. Rooting was improved by increasing gas exchange.	Zarei et al. [91]
BA-1, BA-21, BA-41, BA-49, BA-61, BA-71	Stem segments with two nodes	Callogenesis, shoot proliferation	MS, DKW, WPM, B5, BABI media + TDZ (0.5 μM), 2,4-D (10, 20, 30 μM)	25 °C under a 16 h photoperiod (10–41 ± 4 µmol/m^2^/s)	The maximum shoot regeneration was observed in DKW + 0.5 μM. DKW+ 10 μM 2,4-D was the best treatment for callogenesis.	Page et al. [92]
Hemp cultivar (YUNMA7)	Immature embryo hypocotyls, true leaves, cotyledons and hypocotyls	Indirect shoot organogenesis	Callus induction medium: MS+ 1 mg/L Nicotinic acid + 1 mg/L Pyridoxine-HCl + 10 mg/L Thiamine-HCl + 0.1 g/L Myo-inositol + 3% Sucrose + 2.5 g/L Phytagel + 1 mg/L 2,4-D + 0.25 mg/L Kin + 100 mg/L Casein hydrolysate Regeneration medium: 1/2 strength MS + 1.5% Sucrose + 3.5 g/L Phytagel + 0.5 mg/L TDZ + 0.3 mg/L 6-BA + 0.2 mg/L NAA + 0.2 mg/L IAA Rooting medium: 1/2 strength MS + 1.5% Sucrose + 3.5 g/L Phytagel + 0.2 mg/L NAA + 0.5 mg/L IBA + 0.01 mg/L Zea^RIB^	26 °C under continuous light (50 µmol/m^2^/s)	Over 20% of the immature embryo hypocotyls developed embryogenic calli within 5 days, and the hypocotyls collected 15 days after anthesis (D15) produced more calli (at an average of 31.08%) compared to those collected earlier or later. Throughout the 4-week incubation, the induction frequencies of only 5.97% in true leaves, 7.65% in cotyledons, and 5.31% in hypocotyls were observed. After an additional 2 weeks, proliferating tissues were transferred to the regeneration medium and 6.12% of the D15 calli produced shoots, and less than 3% of the calli developed proliferated shoots from the other three explants.	Zhang et al. [93]

2,4-D: 2,4 dichlorophenoxyacetic acid; BABI: BDS as modified at Arkansas Bioscience Institute; BAP: 6-benzylaminopurine; CBD: cannabidiol; Dicamba: 3,6-dichloro-2-methoxybenzoic acid; DKW: Driver and Kuniyuki Walnut; IAA: indole-3-acetic acid; IBA: indole-3-butyric acid; MS: Murashige and Skoog medium; NAA: 1-naphthaleneacetic acid; NR: not reported; PGR: plant growth regulator; TDZ: thidiazuron; THCA: tetrahydrocannabinolic acid.

**Table 3:** Protoplast isolation studies in Cannabis.

Genotype(s)	Explant	Protoplast Isolation Procedure	References
Cherry x Otto II: Sweetened	Mesophyll of young, not fully expanded leaves of in vitro grown plantlets	Enzymolysis solution composed of 0.3% w/v Macerozyme R-10, 20 mM MES (2-(N-morpholino) ethanesulfonic acid), 1.25% w/v Cellulase R-10, 0.4 M mannitol, 0.1% w/v bovine serum albumin, 10 mM calcium chloride, 20 mM potassium chloride, and 0.075% w/v Pectolyase Y23, adjusted to pH 5.7 and heated to 55 °C for 10 min	Beard et al. [141]
Finola	Etiolated hypocotyls and mesophyll of leaf	Enzyme solution composed of 0.4% Macerozyme R–10 and 1.5% Cellulase Onozuka R-10	Lazič [142]
Mexican strain	Leaf cells	Digestion solution supplemented with 88 mM sucrose, 0.4 M mannitol, 1% (w/v) Cellulase Onozuka R-10, 0.1% (w/v) pectolyase Y-23, and 0.2% (w/v) Macerozyme R-10 at 30 °C for 4 h with gentle agitation	Morimoto et al. [143]

**Table 4:** Polyploidy induction studies in Cannabis.

Genotype(s)	Applied Antimitotic Agent(s)	Polyploidy Induction Efficiency (%)	Survival Rate of Induced Polyploids (%)	Method of Confirmation	Outcomes and Remarks	References
Unspecified Iranian Cultivar	Colchicine (0.1–0.2% w/v)	59.1 for 24 h 42.1 for 48 h	73.33 for 24 h 63.33 for 48 h	Stomate size/density, leaf morphology, and flow cytometry	0.2% colchicine was required to induce polyploidy. Polyploids exhibit wider leaves, larger stomata, and larger male flowers. No effect on cannabinoid production in male and female flowers was reported however female polyploid leaves demonstrated a significant increase in CBD concentration.	Mansouri and Bagheri [162]
THC Dominant Indica and Balanced THC/CBD Indica Dominant Hybrid (Canopy Growth Corp.)	Oryzalin (20–150 µM)	66.7	37.5	Stomate size/density, flow cytometry, and chromosome count	The THC dominant cultivar was only induced under the 40 µM treatment and unsuccessful treatments produced many mixoploids. The balanced cultivar was successfully induced between a range of 20–60 µM. Tetraploid flowers had increased CBD and CBDA content however overall cannabinoid and terpene concentrations were not significantly different. Polyploids exhibit larger leaf area, larger stomata, reduced rooting success, increased sugar leaf trichome density, and decreased stomata density.	Parsons et al. [161]
Hemp cultivars (Youngsim10, Mountain Mango, Cherry Wine, Wife, and Abacus × Wife)	Colchicine (0.02% or 0.05%)	26–64% for 12 h in different cultivars	Not reported	Stomate size/density, leaf morphology, and flow cytometry	0.05% colchicine for 12 h was required to induce polyploidy. Polyploids exhibit thickened hypocotyls and cotyledons, as well as larger stomata.	Kurtz et al. [160]

**Table 5:** *Agrobacterium*-mediated gene transformation studies in Cannabis.

Genotype(s)	Explant(s)	*Agrobacterium* Strain(s)	Additives (mg/L)	Selection Marker	Promoter (s)	CCP (day)	OD (nm)	Transgene(s)	Method of Confirmation	Efficiency (%)	References
Fedora19, Felina34	Shoot tips	NR	CF (NR)	Herbicide	NR	NR	NR	PGIP	Post-inoculation with *Botrytis cinerea.*	≥50	MacKinnon et al. [171]
UnikoB, Kompolti, Anka, Felina-34	Stem, leaf	*A. tumefaciens* EHA101	Spc (150), K (50), AS (100 µM), T (300), D-mannose (1, 2, 3%)	D-mannose, Spc	Ubq3, NOS	3	1.6–1.8 (600)	PMI, Spc	PMI assay, PCR, Southern blot	15.1–55.3	Feeney and Punja [172]
Futura77, Delta405, Delta-llosa, CAN0221, CAN0111	Cotyledonary node, hypocotyls, primary leaves, cotyledons	*A. tumefaciens* LBA4404, C58, IVIA 251, and *A. rhizogenes* 476, 477, 478, A424, AR10GUS, A4, AR10, R1601	AS (20, 100, 200 µM), sucrose (0.5, 2%), sodium citrate (20 mM), MES (30 mM)	K, Carb, Rif	35S::GUS-INT; p35S-CODA-CAMV3′	2	NR	*Bam*HI, *Xho*I, GUS; LBA-*rolABC* (*Eco*RI); LBA-*rolA* (*Eco*RI-*Bam*HI); LBA-*rolB* (*Sma*I-*Hpa*I); LBA-*rolC* (*Hind*IIII-*Eco*RI)	GUS assay, PCR,	43–98 for *A. rhizogenes*; 33.7–63 for *A. tumefaciens*	Wahby et al. [173]
UnikoB, Kompolti, Anka, Felina-34	Stem, leaf	*A. tumefaciens* EHA101	Spc (150), K (50), AS (100 µM),T (300), D-mannose (1, 2, 3%)	D-mannose, Spc	Ubq3, NOS	3	1.6–1.8 (600)	PMI and Spc	PMI assay, PCR, Southern blot	15.1–55.3	Feeney and Punja [174]
Ferimon, Fedora 17, USO31, Felina 32, Santhica 27, Futura 75, CRS-1, CFX-2	male and female flowers, stem, leaf, root	*A. tumefaciens* LBA4404, GV3101, EHA105	200 μM AS, 2% glucose, 10 mM MES, Silwett L-77; Pluronic F-68; L-Ascorbic acid; PVP	CmR	CAMV35s, OCS	3	0.5 (600)	*eGFP*, *uidA*, *att*BI, *att*BII, *CsPDS*, CmR, His^-^	GFP assay, GUS assay, qPCR	10–80	Deguchi et al. [175]
Candida CD-1, Holy Grail x CD-1, Green Crack CBD, Nightingale	Cotyledons, leaves	*A. tumefaciens* EHA105	100 µM AS, 3 mM Silver thiosulfate	K, Rif	CAMV35s, Ubq3, NOS	3	0.6 (600)	PMI, GUS	GUS assay, MUG assay, PCR	45–70.6	Sorokin et al. [176]
Hemp strain (DMG278)	immature embryo hypocotyls	*A. tumefaciens* AGL1	50 mg/L K, 20 mg/L Rif	K, Rif	CaMV 35S promoter and NOS terminator	3	0.1 (600)	*Cs*GRF3*–Cs*GIF1	GFP assay, PCR,	63%	Zhang et al. [93]

NR: not reported; CF: cefotaxime; K = kanamycin; T: Timentin; Spc = spectinomycin; AS: acetosyringone; PGIP: polygalacturase inhibitory proteins; PMI: phosphomannose isomerase; Carb: Carbenicillin; Rif: rifampicin; His^-^: histidine heterotrophy; GUS: β-glucuronidase; MES: 2-N-morpholineethanesulfonic acid; PVP: polyvinylpyrrolidone; CmR: Chloramphenicol; *Cs*GRF3: *Cannabis sative* growth-regulating factor3; *Cs*GIF1: *Cannabis sative* GRF-interacting factor1.

**Table 6:** The main characteristics of some tools for the design of gRNA for genome editing in cannabis.

Features	Tools				
	CRISPOR	CCTop	CHOPCHOP	Breaking Cas	CRISPR RGEN Tools
wtSpCas9 nuclease/orthologues and Cas9 mutants	+/+	+/+	+/+	+/+	+/+
Cpf1 (Cas12a)	+	+	-	+	+
Custom PAM	+	-	+	+	-
nickases/FokI-Cas9	-	-	+/+	-	-
nuclease-deaminase	-	-	-	-	+
(proto)spacer length	-	+	-	+	+
5′-end of gRNA/in vitro transcription promoter	+/-	+/+	+/-	-	-
mismatch	-	+	+	+	+
indels in spacers and protospacers	-	-	-	-	+
GC-content in protospacers	-	+	+	-	-
input of DNA through the clipboard/as a file	+/-	+/+	+/-	+/+	+/+
input of individual genomes vis gene name or Accession Number/input of DNA using the genome coordinates	-/+	-	+/+	-	+/-
multiple sequences	-	+	-	-	-
ranked gRNAs	+	+	+	+	-
off-target sites	+	+	-	+	+
microhomology	+	-	-	-	+
restriction sites	+	-	+	-	-
both DNA strands/edited region (exon, intron, intergenic spacer)	+/+	+/+	+/+	+/+	+/+
presence of the TTT(T) sequence	-	-	-	+	-
GC-content in protospacers/secondary gRNA structure (constant and variable parts)	-	-	+/-	-	+/-
oligonucleotides and primers for cloning/PCR detection	+/+	-	+/-	-	-
demo version	-	-	-	+	-
off-line	+	+	-	-	+

+ and - indicate the existence and lack of the feature, respectively.

## Data Availability

Not applicable.
